# Siplizumab, an Anti-CD2 Monoclonal Antibody, Induces a Unique Set of Immune Modulatory Effects Compared to Alemtuzumab and Rabbit Anti-Thymocyte Globulin *In Vitro*

**DOI:** 10.3389/fimmu.2020.592553

**Published:** 2020-11-11

**Authors:** Christian Binder, Felix Sellberg, Filip Cvetkovski, Erik Berglund, David Berglund

**Affiliations:** ^1^Section of Clinical Immunology, Department of Immunology, Genetics and Pathology, Uppsala University, Uppsala, Sweden; ^2^Research and Development, ITB-Med AB, Stockholm, Sweden; ^3^Department of Clinical Science, Intervention and Technology (CLINTEC), Division of Transplantation Surgery, Karolinska Institute and Karolinska University Hospital, Stockholm, Sweden

**Keywords:** siplizumab, T cell biology, immunotherapy, costimulation blockade, immune modulation

## Abstract

Antibodies are commonly used in organ transplant induction therapy and to treat autoimmune disorders. The effects of some biologics on the human immune system remain incompletely characterized and a deeper understanding of their mechanisms of action may provide useful insights for their clinical application. The goal of this study was to contrast the mechanistic properties of siplizumab with Alemtuzumab and rabbit Anti-Thymocyte Globulin (rATG). Mechanistic assay systems investigating antibody-dependent cell-mediated cytotoxicity, antibody-dependent cell phagocytosis and complement-dependent cytotoxicity were used to characterize siplizumab. Further, functional effects of siplizumab, Alemtuzumab, and rATG were investigated in allogeneic mixed lymphocyte reaction. Changes in T cell activation, T cell proliferation and frequency of naïve T cells, memory T cells and regulatory T cells induced by siplizumab, Alemtuzumab and rATG in allogeneic mixed lymphocyte reaction were assessed *via* flow cytometry. Siplizumab depleted T cells, decreased T cell activation, inhibited T cell proliferation and enriched naïve and *bona fide* regulatory T cells. Neither Alemtuzumab nor rATG induced the same combination of functional effects. The results presented in this study should be used for further *in vitro* and *in vivo* investigations that guide the clinical use of immune modulatory biologics.

## Introduction

Induction therapy is administered peri-transplant to minimize the risk of acute rejection and, ideally, to decrease the need for maintenance immunosuppression (mIS) long-term. Commonly, antibodies are used as part of induction therapy to induce target cell depletion and/or inhibit immune cell activation. Autoimmune conditions are characterized by unwanted immune responses against autologous tissue. Similar to transplantation, monoclonal antibody therapy for treatment of autoimmune conditions aims at depletion and/or inhibition of disease-mediating cells.

Depletion of antibody-bound cells can be mediated through complement-dependent cytolysis (CDC), antibody-dependent cell-mediated cytotoxicity (ADCC), antibody-dependent cell phagocytosis (ADCP), and apoptosis induction. While depletory antibodies have more side effects than non-depletory induction antibodies, they have been shown to be superior in preventing acute rejection (1). Depletion is not undesirable *per se*; however, memory T cells (Tmem) are more resistant to depletion than naïve T cells (Tn) leading to a relative enrichment of Tmem post-depletion (2). Further, irrespective of initial phenotype, T cells assume a memory phenotype when undergoing homeostatic proliferation in lymphopenic environments which further skews the post-depletion T cell repertoire toward a memory phenotype (3). Tmem are more resistant to immunosuppressive medication and have a lower activation threshold than Tn which increases the risk of rejection and decreases the likelihood of allograft tolerance in rodents (4). Moreover, enrichment of Tmem post-transplant is known to be correlated with worse clinical outcomes (4). Theoretically, an optimal depletory antibody agent should selectively deplete alloreactive Tmem while preserving alloreactive regulatory T cells (Tregs) and non-alloreactive T cells.

One form of activation inhibition is costimulatory blockade (CoB). CoB agents have been shown to reduce the risk of acute rejection while having less side-effects compared to commonly used depletory antibodies (5). Siplizumab, a humanized monoclonal anti-CD2 IgG1 antibody, is currently in clinical development after promising immunomodulatory results have been observed *in vitro* (6, 7), in chimpanzees (8), and phase II clinical trials (9). In addition to CD2/LFA3 CoB, siplizumab induces depletion of CD2^+^ cells with previous *in vitro* evidence suggesting ADCC as one mechanism that is elicited by siplizumab as MLR suppression by siplizumab was contingent on the presence of NK cells (7). ADCP may be another mechanism through which siplizumab induces depletion *in vivo*. CD2 expression is increased on Tmem relative to naïve T cells (Tn), and on activated T cells relative to resting T cells (10). Thus, anti-CD2 agents more selectively target alloreactive Tmem. Notably, Lo et al. (10) showed that CD2 and CD28 expression on T cells is inversely correlated, with a fraction of Tmem loosing CD28 expression completely (11). This indicates that CD2 may be a suitable antigen to target in conditions characterized by excessive Tmem activation. Overall, CoB is becoming an important tool which can prevent the activation of allo- and autoreactive T cells and may even divert them into apoptosis, anergy or a Treg phenotype (12, 13).

Alemtuzumab is a humanized monoclonal anti-CD52 IgG1 antibody (also known as CAMPATH-1H) approved for treatment of multiple sclerosis (14). It is frequently used off-label for transplant induction therapy or chronic lymphocytic leukemia. Alemtuzumab induces severe and long-lasting T, NK, and B cell depletion (15), mainly mediated by ADCC with some contribution from CDC of target cells (16). Rabbit anti-thymocyte globulin (rATG) is a polyclonal mixture of IgG antibodies targeting thymocyte antigens approved for prevention and treatment of acute kidney allograft rejection. Off-label uses of rATG include prevention and treatment of allograft rejection in other types of organ transplantation or stem cell transplantation (17). rATG induces T and NK cell depletion through CDC, apoptosis induction and ADCP (17, 18).

The goal of this study was to further explore the mechanistic profile of siplizumab and to contrast its effects in allogeneic mixed lymphocyte reaction with Alemtuzumab and rATG.

## Methods

### Test Substances

Rabbit anti-thymocyte globulin (Polyclonal anti-thymocyte antigen IgG mixture; Thymoglobuline, Sanofi Genzyme, Cambridge, USA) and Alemtuzumab (humanized anti-CD52 IgG1κ; Lemtrada, Sanofi Genzyme, Cambridge, USA) were purchased from Apoteket Hjärtat AB (Solna, Sweden). Siplizumab (humanized anti-CD2 IgG1κ; ITB-Med, Stockholm, Sweden) is an investigational drug and was provided by the manufacturer.

### Fcγ Receptor Reporter Bioassays

Jurkat effector cells expressing Fcγ receptor I (FcγRI), FcγRIIA, or FcγRIIIA (Promega Corp., Madison, USA) were resuspended in assay buffer 3% ultra-low IgG FBS (Gibco, Thermo Fisher Scientific Inc., Waltham, USA) in RPMI-1640 ATCC Modification (ATCC Mod.; Gibco, Thermo Fisher Scientific Inc., Waltham, USA) and added to white cell culture plates at a final concentration of 1.1 × 10^6^ effector cells per ml once all reagents had been added. Serial dilutions of siplizumab or Fc-silent siplizumab (negative control) in assay buffer or pure assay buffer (untreated controls) were added at the indicated concentrations. The reaction mixtures (final volume 90 µl) were incubated at 37°C 5% CO_2_ for 23 h. After equilibration to room temperature (RT; 22–23°C), 70 µl of freshly thawed Bio-Glo Luciferase assay substrate (Promega Corp., Madison, USA) was added. After 15–20 min of incubation at RT and in the dark, luminescence was measured using a Synergy HTX Multi-Mode plate reader (Biotek Instruments Inc., Winooski, USA). Jurkat reporter cells express no or very low levels of luciferase in the absence of target-bound antibodies. If reporter cells bind a target-bound IgG antibody with their respective FcγR, downstream NFAT signaling induces luciferase expression. Bio-Glo Luciferase assay substrate solution induces cell lysis and contains luciferase substrate. Therefore, increased luminescence indicates agonistic FcγR signaling.

### Isolation of Peripheral Blood Mononuclear Cells

Peripheral blood mononuclear cells (PBMCs) were isolated *via* Ficoll^®^ Paque Plus (GE Healthcare, Chicago, USA) density gradient centrifugation from buffy coats. Buffy coats were obtained from anonymous healthy donors *via* Uppsala University Hospital blood bank (Uppsala, Sweden) or Karolinska University Hospital blood bank (Stockholm, Sweden) and PBMC isolation was carried out within 24 h of blood collection.

### Flow-Cytometric Complement-Dependent Cytolysis (CDC) Assay

PBMC were isolated *via* density gradient centrifugation and incubated with siplizumab or positive control rat anti-CD2 IgG2b mAb (BTI-322, BioTransplant Inc, MA, USA) in 10% ultra-low IgG FBS in PBS at the indicated concentrations for 30 min at 1 × 10^7^ cells per ml at RT with shaking. Subsequently, an equal volume of rabbit complement (inno-train Diagnostik GmbH, Kronberg, Germany) was added followed by 60 min of incubation at RT with shaking. Cells were subsequently washed twice in saline solution, blocked with Fc-receptor binding inhibitor (Invitrogen; Thermo Fisher Scientific Inc., Waltham, USA) and then stained with 7-Aminoactinomycin D (7-AAD; Invitrogen, Thermo Fisher Scientific Inc., Waltham, USA), anti-CD3 APC (Clone HIT3a), anti-CD56 BV421 (Clone NCAM16.2), and anti-CD19 APC-H7 (Clone SJ25-C1) for 30 min on ice and shielded from light. All antibodies were purchased from BD Biosciences (San Diego, USA). Subsequently, samples were washed twice in saline solution and acquired using a BD Celesta flow cytometer (BD Biosciences, San Diego, USA). Positive staining of lymphocytes for 7-AAD was used as a read-out for complement-dependent cytolysis (CDC; **Supplementary Figure S1**). Post-acquisition editing and data analysis was conducted using FlowJo^®^ 10.5.3 software (FlowJo LLC, Ashland, USA).

### Allogeneic Mixed Lymphocyte Reaction (MLR)

Equal amounts of PBMC from each donor were mixed in PBS at a concentration of 1.5–2.0 × 10^7^ cells per ml and stained with violet proliferation dye 450 (VPD450; BD Biosciences, San Diego, USA) according to the manufacturer’s instructions. This study employed a two-way allogeneic MLR setup where PBMC from neither donor were inactivated *via* irradiation or chemical treatment. This means that PBMC from both donors functioned as responders to foreign HLA on PBMC from the other donor and stimulators providing foreign HLA against which PBMC from the other donor can respond. As this study investigated the effect of antibody agents on T and NK cell responses in general and not responsiveness of a specific donor, this setup was deemed satisfactory.

For Treg analysis, stained PBMC were washed and resuspended in 10% heat-inactivated fetal bovine serum (FBS; Gibco, Thermo Fisher Scientific Inc., Waltham, USA) in RPMI-1640 ATCC Mod. supplemented with 50 U/ml Streptomycin and Penicillin (Gibco, Thermo Fisher Scientific Inc., Waltham, USA). Resuspended PBMC were dispensed into round-bottom 96-well cell culture plates (for flow cytometry) and pure medium (no antibody controls) or antibody solution (final concentration 10 µg/ml) in culture medium was added to a final concentration of 2 × 10^6^ cells per ml (final volume 200 µl). For Treg phenotyping cells were kept in culture at 37°C, 5% CO_2_ for 7, 10, and 14 days with 100 µl fresh culture medium (no additional antibody; final volume = 300 µl) being added after 7 days of culture.

For all other MLRs, stained PBMC were washed and resuspended in 10% heat-inactivated FBS (Gibco, Thermo Fisher Scientific Inc., Waltham, USA) in AIM V medium (Gibco, Thermo Fisher Scientific Inc., Waltham, USA). Resuspended PBMC were dispensed into round-bottom 96-well cell culture plates (for flow cytometry) and pure medium (no antibody controls) or antibody solution (final concentration 0.0001, 0.001, 0.01, 0.1, 1, or 10 µg/ml) was added to a final concentration of 2 × 10^6^ cells per ml (final volume 200 µl). MLRs were incubated at 37°C, 5% CO_2_ for 1, 2, 4, and 7 days, respectively. On day 6, 100 µl fresh culture medium (no additional antibody; final volume = 300 µl) was added to each well.

### Pure T Cell Culture

T cells were isolated from PBMC using the Pan T cell Isolation Kit (Miltenyi, Bergisch Gladbach, Germany) according to the manufacturer’s instructions. T cells were kept in autologous culture at a concentration of 1 × 10^6^ cells/ml without addition of antibody (untreated controls) or addition of 10 µg/ml of the respective antibody agent (All other culture conditions are as described in MLR section above for Treg analysis). Cells were analyzed *via* flow cytometry prior to culture and on days 1, 3, 5, and 7.

### Target Antigen Expression

Resting PBMC were stained and analyzed for target antigen expression (See below). Subsequently, PBMC in autologous culture were activated *via* CD28/CD3 beads (Miltenyi, Bergisch Gladbach, Germany) for 48 h (bead to cell ratio 5:1; other culture conditions same as in MLR) and target antigen expression was investigated.

### Flow Cytometry

Before staining, cells were washed twice in saline solution. All antibodies listed below were purchased from BD Biosciences (San Diego, USA) if not specified otherwise.

Samples for analysis of T cell activation, T cell proliferation, T cell subpopulations and NK cell activation were blocked with Fc-receptor binding inhibitor (Invitrogen; Thermo Fisher Scientific Inc., Waltham, USA) and then stained with anti-CD3 VioGreen (Miltenyi, Bergisch Gladbach, Germany; Clone REA613), anti-CD45RA BV650 (Clone HI100), anti-CD69 BV786 (Clone FN50), anti-CD8 BB550 (Clone RPA-T8), anti-CD56 PE (Miltenyi; Clone REA196), anti-HLA-DR PerCP-Cy5.5 (Clone G46-6), anti-CCR7 APC (Clone G43H7), and anti-CD4 APC-Vio770 (Miltenyi; Clone REA623). T and NK cells were defined as CD3^+^ CD56^−^ and CD3^−^ CD56^+^, respectively. CD69+ and HLA-DR+ T cells were identified using fluorescence minus one controls (**Supplementary Figure S2**). T cell subpopulations were defined as follows: Naïve T cells: CCR7^+^ CD45RA^+^; Central memory T cells: CCR7^+^ CD45RA^−^; Effector memory T cells: CCR7^−^ CD45RA^−^; Terminally differentiated effector memory T cells: CCR7^−^ CD45RA^+^ (**Supplementary Figure S3**). Cell proliferation was assessed using VPD450 (VPD450high: Non-proliferated; VPD450low: Proliferated). Samples were stained in the dark at 4°C and washed twice in saline solution followed by acquisition using a BD Celesta flow cytometer (BD Biosciences, San Diego USA). MLR samples were phenotyped *via* flow cytometry at baseline (day 0), days 1, 2, 4, and 7.

Samples for Treg analysis were stained anti-CD4 FITC (Clone RPA-T4), anti-CD25 PE (Clone M-A251), anti-CD45RA APC-H7 (Clone HI100), anti-CD127 PerCP-Cy5.5 (Clone HIL-7R-M21), and anti-FoxP3 Alexa647 (Clone 259D/C7). They were permeabilized using BD Human FoxP3 Buffer Set (BD BioSciences, San Diego, USA) according to the vendor’s protocol. Tregs were identified as CD4^+^ CD127^−^ CD25^+^ FoxP3^+^ (**Supplementary Figure S4**).

Samples from Pure T cell cultures were analyzed with anti-CD69 APC (Clone FN50) and anti-CD3 PerCP-Cy5.5 (Clone SP34-2).

Samples investigating antigen expression were incubated with Fc-receptor binding inhibitor (Invitrogen; Thermo Fisher Scientific Inc., Waltham, USA) and then washed twice in FBS staining buffer (BD BioSciences, San Diego, USA) followed by incubation with 10 µg/ml siplizumab, Alemtuzumab or rATG for 20 min. Unbound antibody was removed before staining with secondary anti-human IgG Fc BV421 (Biolegend, San Diego, USA; Clone HP6017). Unbound antibody was removed before surface staining for T cell subpopulations, regulatory T cells or B and NK cells. T cell subpopulation and Treg panels were the same as described above. NK/B cell panel consisted of anti-CD16 FITC (Clone 3G8), anti-CD56 PE (Miltenyi, Bergisch Gladbach, Germany; Clone REA196), anti-CD3 APC (Clone SK7) anti-CD14 APC-H7 (Clone M5E2), and anti-CD20 PerCP (Clone SJ25C1). MFI values for T and NK cell populations were adjusted for background signal using an isotype control. More specifically, the MFI value observed for each T/NK cell population in PBMC stained with Rituximab and secondary antibody were subtracted from values of samples incubated with siplizumab, Alemtuzumab or rATG.

Treg analysis samples, pure T cell culture samples and antigen expression samples were stained in the dark at 4°C and were acquired using a FACSVerse flow cytometer (BD Biosciences, San Diego, USA). MLR samples were phenotyped *via* flow cytometry on days 7, 10, and 14 for Treg analysis. Cell proliferation was assessed using VPD450 (VPD450high: Non-proliferated; VPD450low: Proliferated).

Post-acquisition editing and data analysis of all flow cytometry data was conducted using FlowJo^®^ 10.5.3 software (FlowJo LLC, Ashland, USA).

### FoxP3 Methylation Analysis

After 7 and 14 days of MLR as described above—supplemented with no antibody (Untreated control) or 10 µg/ml of the respective antibody agent—CD4^+^ CD25^+^ FoxP3^+^ cells were sorted *via* fluorescent-activated cell sorting (FACS) using a FACSAria III flow cytometer (BD BioSciences, San Diego, USA). Samples were frozen in liquid nitrogen as minimal fluid pellets and stored at −80°C until analysis by EpigenDx (Hopkinton, Massachusetts USA) using direct bisulfite modification with pyrosequencing (Location: Base pairs −2,330 to −2,263 relative to initiation codon ATG).

### Graphs and Statistical Analysis

Visualization of results and statistical analysis of underlying data were carried out using GraphPad Prism 8 software (GraphPad Software, San Diego, USA). Data displayed in graphs and flow cytometry gating strategies can be found in supplementary information. Data were analyzed using two-way ANOVA followed by Dunnett’s multiple comparison test with no antibody controls serving as the comparison data set. Significant differences are indicated as * (p < 0.05) in figures.

## Results

### Fcγ Receptor Reporter Bioassays and Complement-Dependent Cytotoxicity (CDC)

**Figure 1:**
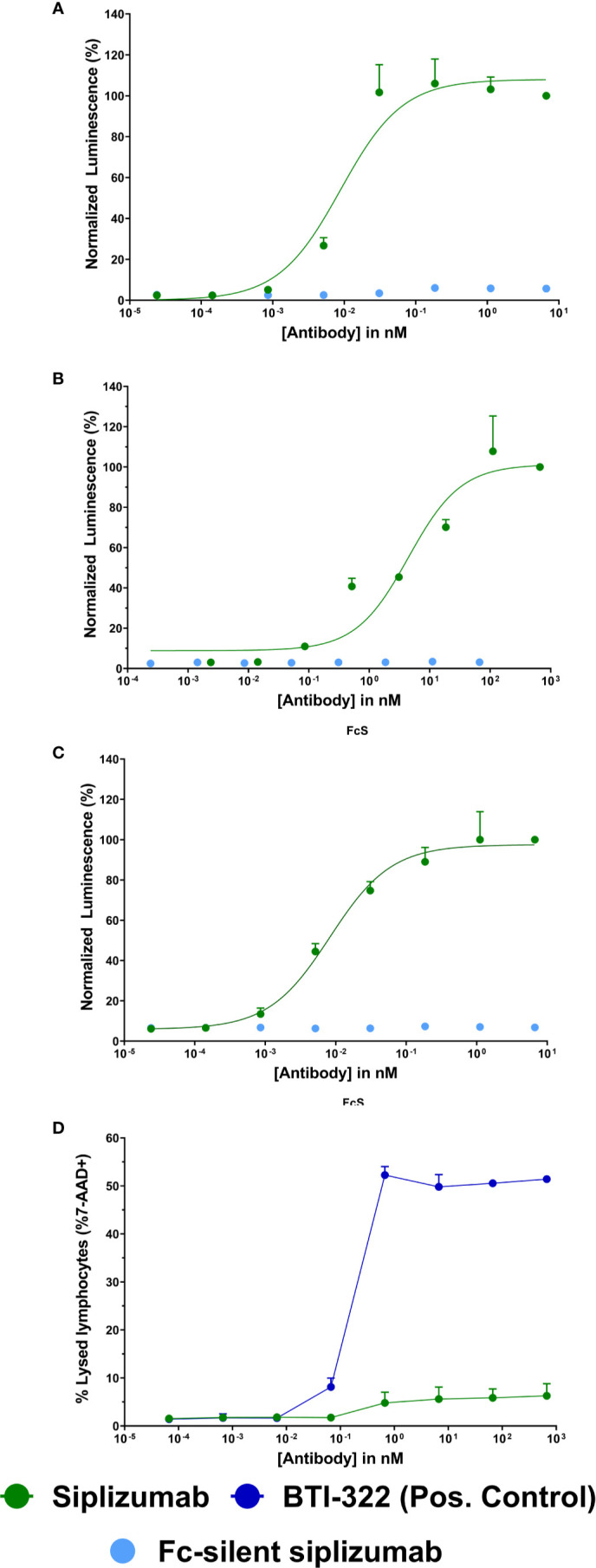
Fc γ receptor (FcγR) signaling and complement-dependent cytolysis (CDC) assays Jurkat reporter cells (Promega) stably expressing Fc γ receptor (FcγR) I **(A)**, FcγRIIA **(B)**, or FcγRIIIA **(C)**, respectively, incubated with increasing concentrations of siplizumab or Fc-silent siplizumab. Reporter cells binding the Fc-fragment of a target-bound IgG antibody with their FcγR induced expression of a luciferase reporter gene resulting in a luminescence signal upon addition of assay substrate (Promega). Luminescence was normalized to highest concentration of siplizumab and is displayed as mean percentage ± SD (N = 3). **(D)** CDC of PBMC mixed with different doses of siplizumab or positive anti-CD2 control (BTI-322). Data displayed as mean percentage ± SD (N = 2).

As displayed in **Figures 1A–C**, siplizumab bound to and induced signaling through all FcγRs. In contrast, Fc-silent siplizumab induced no FcγR-mediated signaling (**Supplementary tables S1-S3**). CD2 binding comparable to siplizumab and absence of FcγR binding by Fc-silent siplizumab was confirmed *via* surface plasmon resonance (data not shown). While siplizumab did not induce CDC, the positive control anti-CD2 mAb (BTI-322) induced a dose-dependent increase in the percentage of 7-AAD+ lymphocytes (**Figure 1D** and **Supplementary Table S4**).

### T Cell Proliferation

**Figure 2:**
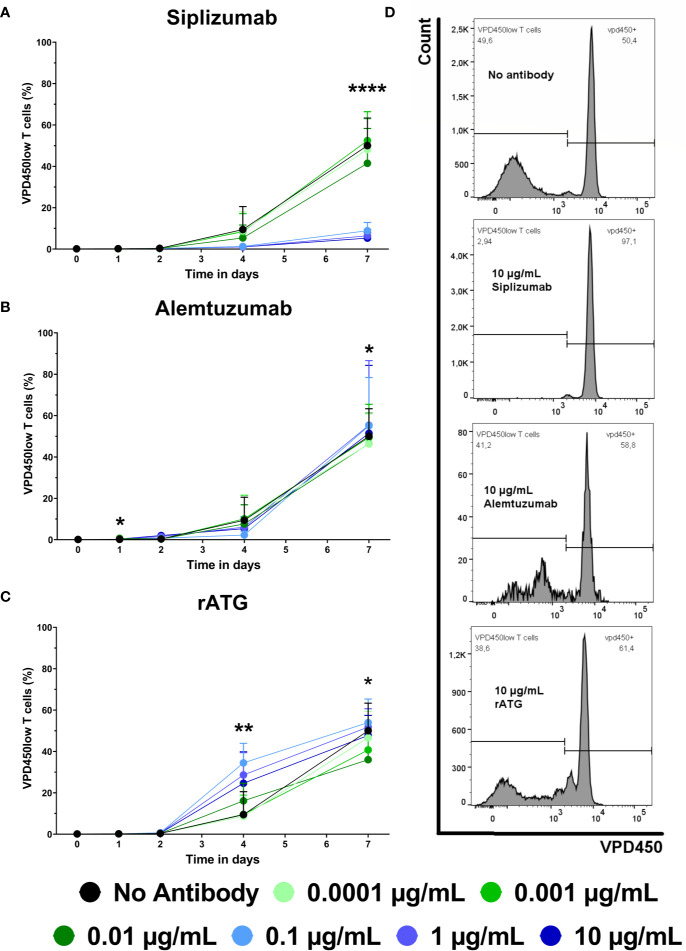
The effect of siplizumab **(A)**, Alemtuzumab **(B)**, and rabbit anti-thymocyte globulin (rATG) **(C)** on T cell proliferation in allogeneic mixed lymphocyte reaction (MLR). T cell proliferation was measured at baseline (day 0) and on days 1, 2, 4, and 7. Data is displayed as the mean of all data points (N = 9 donor pairs) ± SD. T cells were identified as CD3^+^ CD56^−^ lymphocytes. Proliferation was measured *via* violet proliferation dye 450 (VPD450; VPD450low = proliferated). Data were analyzed using two-way ANOVA followed by Dunnett’s multiple comparison test with untreated controls (no antibody) serving as the comparison data set (*p < 0.05, **p < 0.01, ****p < 0.0001). **(A)** Siplizumab did not affect T cell proliferation up to day 4. After 7 days of allogeneic MLR, siplizumab significantly inhibited T cell proliferation at 0.01 µg/ml (p = 0.0358), 0.1, 1, and 10 µg/ml (p < 0.0001). **(B)** Alemtuzumab did induce notable changes in T cell proliferation. **(C)** rATG significantly increased T cell proliferation after 4 days of allogeneic MLR at 0.01 µg/ml (p = 0.0019) and 1 µg/ml (p = 0.0150) and significantly decreased T cell proliferation after 7 days of allogeneic MLR at 0.01 µg/ml (p = 0.0252). **(D)** Representative T cell proliferation histograms from MLRs treated with no antibody, 10 µg/ml siplizumab, 10 µg/ml Alemtuzumab, and 10 µg/ml rATG.

Proliferation of alloreactive T cells can contribute to allograft rejection in transplant patients. Therefore, the effect of siplizumab, Alemtuzumab and rATG on T cell proliferation in allogeneic MLR was investigated. T cell proliferation was assessed *via* violet proliferation dye 450 (VPD450) tracking as measured by flow cytometry after 1, 2, 4, and 7 days of allogeneic MLR (**Figure 2** and **Supplementary Table S5**). Siplizumab significantly reduced T cell proliferation after 7 days of allogeneic MLR at 0.01 µg/ml (p = 0.0358), 0.1, 1, and 10 µg/ml (p < 0.0001). Alemtuzumab did not meaningfully affect T cell proliferation at any time point. rATG significantly increased T cell proliferation after 4 days of allogeneic MLR at 0.01 µg/ml (p = 0.0019) and 1 µg/ml (p = 0.0150). Further, rATG significantly decreased T cell proliferation after 7 days of allogeneic MLR at 0.01 µg/ml (p = 0.0252).

### Antibody-Mediated Cell Depletion

**Figure 3:**
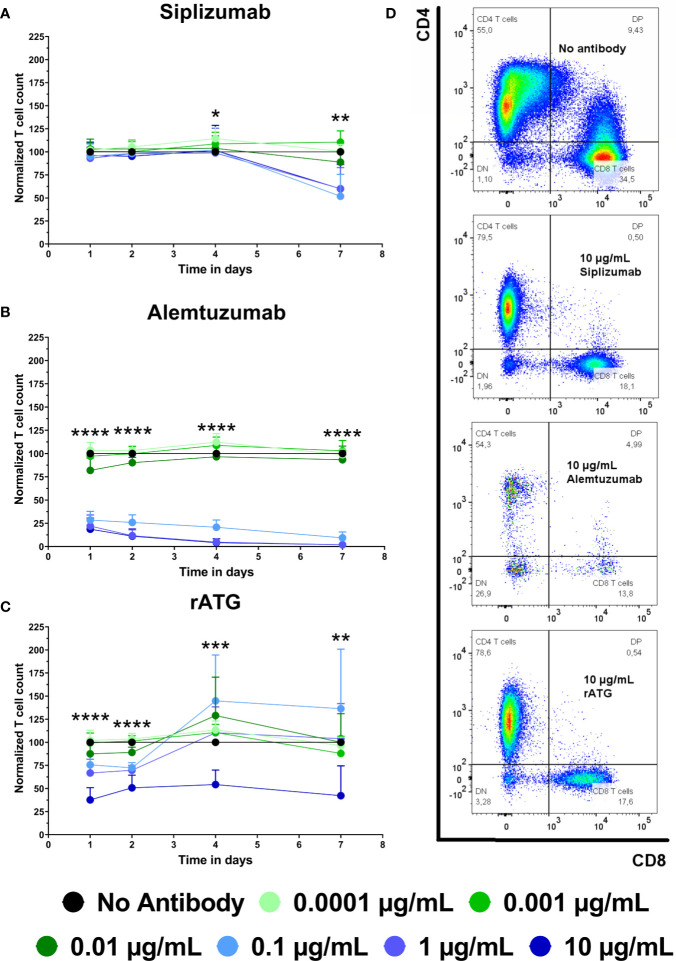
The effect of siplizumab **(A)**, Alemtuzumab **(B)**, and rabbit anti-thymocyte globulin (rATG) **(C)** on T cell count in allogeneic mixed lymphocyte reaction (MLR). T cell count was measured on days 1, 2, 4, and 7. Data was normalized to T cell count in no antibody control and is displayed as the mean of all data points (N = 9 donor pairs) ± SD. T cell count was measured as number of events in the CD3^+^ CD56^−^ lymphocyte gate. Data were analyzed using two-way ANOVA followed by Dunnett’s multiple comparison test with untreated controls (no antibody) serving as the comparison data set (*p < 0.05, **p < 0.01, ***p < 0.001, ****p < 0.0001). **(A)** Siplizumab did not change normalized T cell count on days 1 to 4 of allogeneic MLR but significantly reduced T cell count after 7 days at 0.1–10 µg/ml (p ≤ 0.0082). **(B)** Alemtuzumab significantly reduced T cell count on all time points at 0.1, 1, and 10 µg/ml (p < 0.0001). At 0.01 µg/ml Alemtuzumab induced significant T cell depletion after 1 (p = 0.0157) and 2 (0.0036) days of allogeneic MLR. **(C)** After 1 and 2 days of allogeneic MLR, rATG induced significant T cell depletion at 0.01 µg/ml (p = 0.0366; p = 0.0018), 0.1 µg/ml (p = 0.0011; p < 0.0001), 1 µg/ml (p = 0.0007; p < 0.0001) and 10 µg/ml (p < 0.0001). Only 10 µg/ml rATG led to sustained depletion after 4 (p = 0.0001) and 7 (p = 0.003) days. **(D)** Representative dot plots illustrating differences in T cell count in MLRs treated with no antibody, 10 µg/ml siplizumab, 10 µg/ml Alemtuzumab, and 10 µg/ml rATG.

To complement proliferation data, T cell count was analyzed (**Figure 3** and **Supplementary Table S6**). T cell count was measured as CD3^+^ CD56^−^ events in the lymphocyte gate and was normalized to untreated controls (No antibody). Siplizumab significantly reduced T cell count after 7 days of allogeneic MLR at 0.1 µg/ml (p = 0.0014), 1 µg/ml (p = 0.0035), and 10 µg/ml (p = 0.0082). Alemtuzumab induced significant T cell depletion on all time points at 0.1, 1, and 10 µg/ml (p < 0.0001). At 0.01 µg/ml Alemtuzumab induced mild but significant T cell depletion after 1 (p = 0.0157) and 2 (0.0036) days of allogeneic MLR. rATG induced significant T cell depletion after one and 2 days of allogeneic MLR at 0.01 µg/ml (p = 0.0366; p = 0.0018), 0.1 µg/ml (p = 0.0011; p < 0.0001), 1 µg/ml (p = 0.0007; p < 0.0001), and 10 µg/ml (p < 0.0001). Only 10 µg/ml rATG led to sustained depletion through days 4 (p = 0.0001) and 7 (p = 0.0030).

### T Cell Subpopulation Distribution

Expansion of alloreactive Tmem can facilitate allograft rejection, especially since they have a lower activation threshold and decreased sensitivity to immunosuppressive agents. Further, Tregs are commonly seen as promoting peripheral tolerance. To investigate whether mAb-induced changes in T cell proliferation and count influenced T cell phenotype, T cell subpopulations were analyzed with regards to naïve, memory and regulatory phenotypes using flow cytometry. Naïve T cells (Tn) were defined as CCR7^high^ CD45RA^+^ and were investigated at baseline and after 1, 2, 4, and 7 days of allogeneic MLR. An increase in Tn signifies a proportional decrease in Tmem subpopulations (Tcm CCR7^high^ CD45RA^−^, Tem CCR7^low^ CD45RA^−^, Temra CCR7^low^ CD45RA^+^). Tregs were identified as CD4^+^ CD127^−^ CD25^+^ FoxP3^+^ and were analyzed after 7, 10, and 14 days of allogeneic MLR.

**Figure 4:**
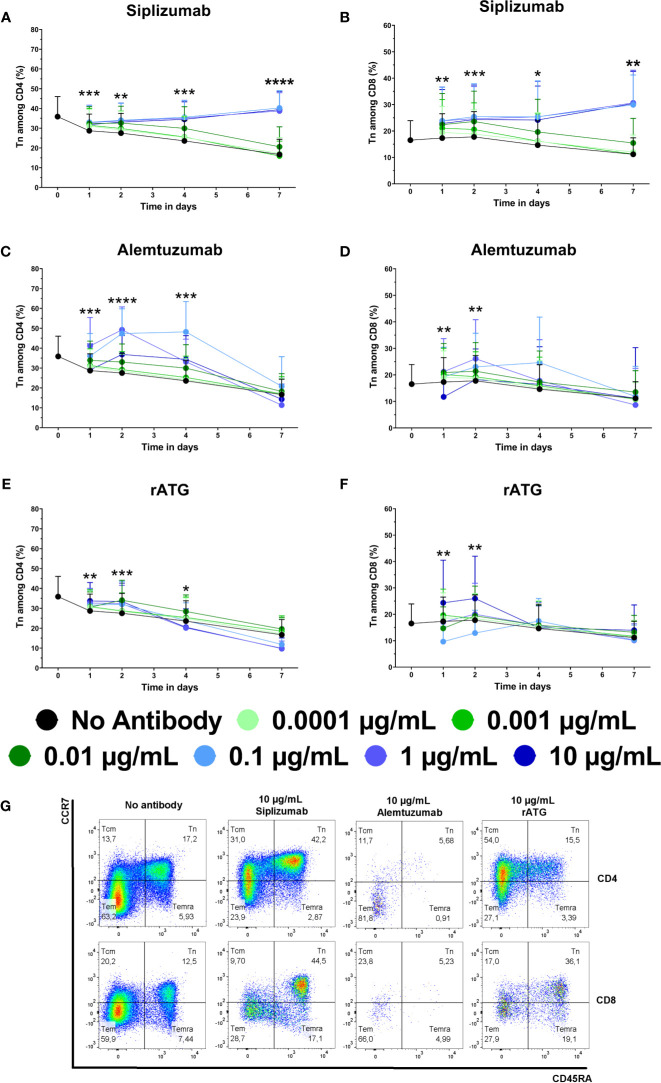
The effect of siplizumab **(A, B)**, Alemtuzumab **(C, D)**, and rabbit anti-thymocyte globulin (rATG) **(E, F)** on naïve CD4 T cell (Tn) enrichment in allogeneic mixed lymphocyte reaction (MLR). T cell subpopulations were analyzed at baseline (day 0) and on days 1, 2, 4, and 7. Data is displayed as the mean of all data points (N = 9 donor pairs) ± SD. CD4 and CD8 T cells were identified as CD3^+^ CD56^−^ CD4^+^ and CD3^+^ CD56^−^ CD8^+^ lymphocytes, respectively. Tn were identified as CD45RA^+^ CCR7^high^. Data were analyzed using two-way ANOVA followed by Dunnett’s multiple comparison test with untreated controls (no antibody) serving as the comparison data set (*p < 0.05, **p < 0.01, ***p < 0.001, ****p < 0.0001). **(A)** Siplizumab enriched CD4+ Tn at all tested concentrations after 1 day of allogeneic MLR (p ≤ 0.0342) as well as at 0.001–10 µg/ml after 2 (p ≤ 0.0077) and 4 (p ≤ 0.0187) days. After 7 days, statistically significant enrichment of CD4+ Tn was observed in samples treated with 0.1 µg/ml (p = 0.0423) and at 0.1–10 µg/ml (p < 0.0001) siplizumab. **(B)** Siplizumab enriched CD8+ Tn at all tested concentrations after 1 day (p ≤ 0.0398), at or above 0.001 µg/ml after 2 days (p ≤ 0.0039), at or above 0.01 µg/ml after 4 days (p ≤ 0.019) and at 0.1 µg/ml or higher after 7 days (p ≤ 0.0044) of allogeneic MLR. **(C)** Alemtuzumab increased CD4+ Tn at 0.0001–1 µg/ml on day 1 (p ≤ 0.0362), 0.01–10 µg/ml on day 2 (p ≤ 0.0255), and 0.01–0.1 µg/ml on day 4 (p ≤ 0.0005) of allogeneic MLR. No statistically significant enrichment of CD4 Tn by Alemtuzumab was detected on day 7. **(D)** No notable enrichment of Tn among CD8 T cells was detected in Alemtuzumab-treated samples. **(E)** rATG enriched CD4+ Tn at 0.001 µg/ml (p = 0.0411) and 0.1–10 µg/ml (p ≤ 0.0285) on day 1, at 0.01–10 µg/ml (p = 0.0411) on day 2 (p ≤ 0.0338), and at 0.01 µg/ml on day 4 (p = 0.0470) of allogeneic MLR. Moreover, 10 µg/ml rATG induced a significant depletion of CD4+ Tn on day 7 (p = 0.0273). **(F)** No clear dose-dependent enrichment or depletion of CD8+ Tn occurred in rATG-treated samples. **(G)** Representative dot plots illustrating differences in subpopulation distribution among CD4 and CD8 T cells in MLRs treated with no antibody, 10 µg/ml siplizumab, 10 µg/ml Alemtuzumab, and 10 µg/ml rATG. Tcm, central memory T cells, CD45RA^−^ CCR7^high^; Tn, naïve T cells, CD45RA^+^ CCR7^high^; Temra, CD45RA^+^ CCR7^low^, effector memory CD45RA+ T cells; tem, Effector memory T cells, CD45RA^−^ CCR7^low^.

Untreated controls (no antibody) tended to display a continuous decline in the frequency of Tn among CD4 and CD8 T cells (**Figure 4** and **Supplementary Tables S7 and S8**). In contrast, siplizumab enriched CD4^+^ Tn at all tested concentrations after 1 day of allogeneic MLR (p ≤ 0.0342) as well as at 0.001–10 µg/ml after 2 (p ≤ 0.0077) and 4 (p ≤ 0.0187) days of allogeneic MLR. After 7 days, statistically significant enrichment of CD4^+^ Tn was observed in samples treated with 0.01 µg/ml (p = 0.0423) and especially at 0.1–10 µg/ml (p < 0.0001) siplizumab. Similarly, siplizumab enriched CD8^+^ Tn at all tested concentrations after 1 day (p ≤ 0.0398), at or above 0.001 µg/ml after 2 days (p ≤ 0.0039), at or above 0.01 µg/ml after 4 days (p ≤ 0.019) and at 0.1 µg/ml or higher after 7 days (p ≤ 0.0044) of allogeneic MLR. Notably, enrichment of Tn by siplizumab at concentrations of 0.1 µg/ml or higher tended to be more pronounced than at 0.01 µg/ml. In comparison, Alemtuzumab increased CD4^+^ Tn at 0.0001–1 µg/ml on day 1 (p ≤ 0.0362), 0.01–10 µg/ml on day 2 (p ≤ 0.0255), and 0.01–0.1 µg/ml on day 4 (p ≤ 0.0005) of allogeneic MLR. No notable enrichment of Tn among CD8 T cells was detected in Alemtuzumab-treated samples. Further, rATG enriched CD4^+^ Tn at 0.001 µg/ml (p = 0.0411) and 0.1–10 µg/ml (p ≤ 0.0285) on day 1, at 0.01–10 µg/ml (p = 0.0411) on day 2 (p ≤ 0.0338), and at 0.01 µg/ml on day 4 (p = 0.0470) of allogeneic MLR. Moreover, 10 µg/ml rATG induced a significant depletion of CD4^+^ Tn on day 7 (p = 0.0273). No clear dose-dependent enrichment or depletion of CD8^+^ Tn occurred in rATG-treated samples.

**Figure 5:**
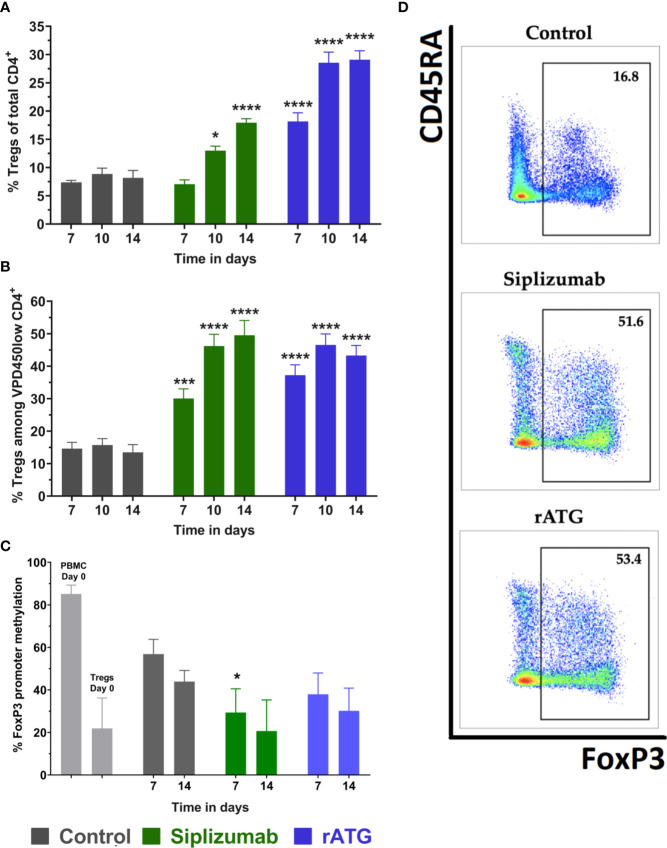
Regulatory T cells (Tregs) in allogeneic mixed lymphocyte reaction (MLR). Cells were harvested and phenotyped after 7, 10, or 14 days of allogeneic MLR. Each antibody was used at a concentration of 10 µg/ml. Data displayed as mean ± SD. Data were analyzed using two-way ANOVA followed by Dunnett’s multiple comparison test (*p < 0.05, ***p < 0.001, ****p < 0.0001) with no antibody controls serving as the comparison data set. Tregs were identified as CD4^+^ CD127^−^ CD25^+^ FoxP3^+^ **(A)** Tregs as % of CD4^+^ T cells over time (N = 12 donor pairs). Siplizumab significantly enriched Tregs among CD4^+^ T cells after 10 (p = 0.0340) and 14 (p < 0.0001) days of allogeneic MLR. Similarly, rATG increased the percentage of Tregs among CD4^+^ T cells on all time points (p < 0.0001 for all time points). **(B)** Percentage of Tregs among proliferated (VPD450low) CD4^+^ T cells (N = 12 donor pairs). Siplizumab (p = 0.0004; p < 0.0001; p < 0.0001) and rATG (p = 0.0007; p < 0.0001; p < 0.0001) significantly enriched Tregs among proliferated CD4^+^ T cells after 7, 10, and 14 days of allogeneic MLR. **(C)** FoxP3 promoter methylation status of regulatory T cells (N = 5 donor pairs) with Tregs from no antibody controls serving as the comparison data set. PBMC were used as a control. Lower percentage methylation indicates higher commitment to Treg phenotype. FoxP3 promoter methylation was significantly decreased in Tregs isolated from siplizumab-treated samples relative to Tregs isolated from no antibody controls after 7 days of allogeneic MLR (p = 0.0190). A decrease trending toward significance (p = 0.0529) was measured in siplizumab-treated samples after 14 days of allogeneic MLR. rATG failed to induce a significant change in FoxP3 promoter methylation, although decreases observed after 7 days trended toward significance (p = 0.0520). **(D)** Representative dot plots illustrating enrichment of Tregs among proliferated CD4 T cells in samples treated with 10 µg/ml siplizumab or 10 µg/ml rATG after 14 days of MLRs.

Untreated controls (no antibody) displayed approximately 7.5% Tregs among total CD4^+^ T cells and 15% among proliferated CD4^+^ T cells on all time points (**Figures 5A, B, D** and **Supplementary Table S9**). Siplizumab induced a significant enrichment of Tregs among total CD4^+^ after 10 (p = 0.0340) and 14 (p < 0.0001) days as well as among proliferated CD4^+^ T cells after 7 (p = 0.0004), 10 (p < 0.0001) and 14 (p < 0.0001) days of allogeneic MLR. Treg phenotyping could not be performed in Alemtuzumab-treated samples due to an insufficient number of Treg events upon flow cytometric analysis (severe T cell depletion). Similar to siplizumab, rATG significantly enriched Tregs among total CD4^+^ T cells and proliferated CD4^+^ T cells after 7 (p < 0.0001; p = 0.0007), 10 (p < 0.0001 for both), and 14 (p < 0.0001 for both) days of allogeneiec MLR.

To further investigate the identity of Tregs (*bona fide* Tregs vs. activation-induced FoxP3 expression), FoxP3 promoter methylation in FACS-sorted Tregs was measured at baseline (day 0) as well as after 7 and 14 days of allogeneic MLR (**Figure 5C** and **Supplementary Table S9**). Higher FoxP3 methylation indicates a higher degree of activation-induced FoxP3 expression. Lower FoxP3 promoter methylation indicates that analyzed Tregs are *bona fide* (phenotypically committed) Tregs. FoxP3 promoter methylation in Tregs isolated from siplizumab-treated samples after 7 days of allogeneic MLR was significantly lower than in Tregs isolated from untreated controls (p = 0.0190). Further, the decrease in FoxP3 promoter methylation measured in Tregs isolated from siplizumab-treated samples after 14 days of MLR trended toward significance (p = 0.0523). In contrast, rATG did not induce a significant decrease in FoxP3 promoter methylation after 7 and 14 days of MLR.

### Activation Marker Expression on T Cells

**Figure 6:**
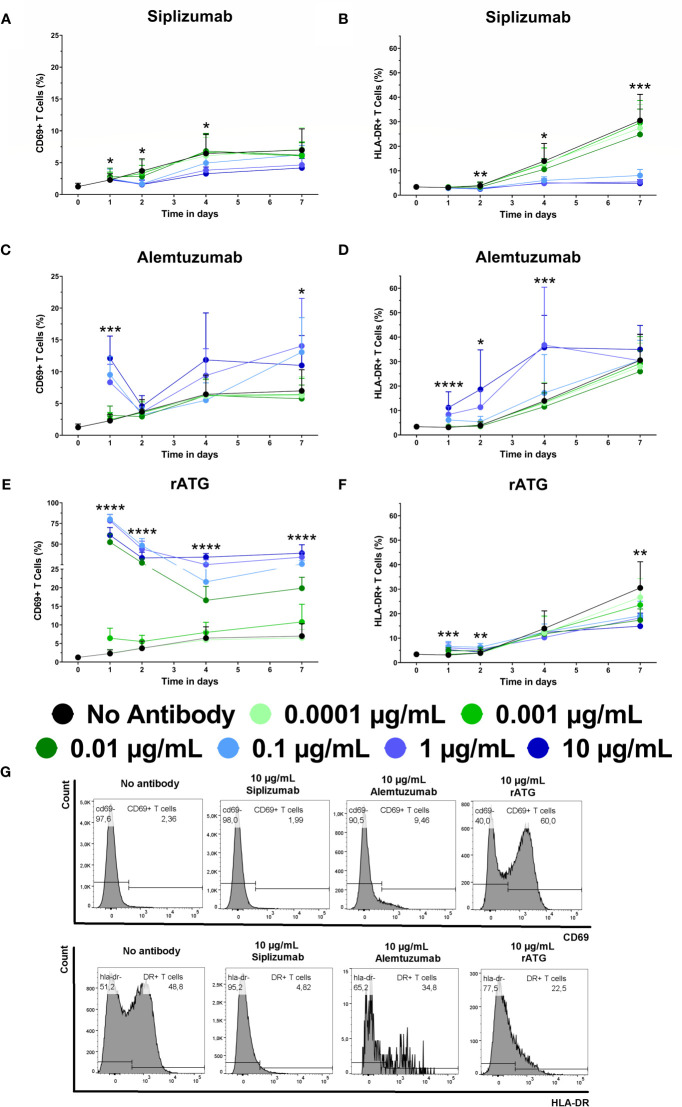
The effect of siplizumab **(A, B)**, Alemtuzumab **(C, D)** and rabbit anti-thymocyte globulin (rATG; E, F) on CD69 and HLA-DR expression on T cells in allogeneic mixed lymphocyte reaction (MLR). Expression of CD69 and HLA-DR were analyzed at baseline (day 0) and on days 1, 2, 4, and 7. Data is displayed as the mean of all data points (N = 9 donor pairs) ± SD. T cells were identified as CD3^+^ CD56^−^. Data were analyzed using two-way ANOVA followed by Dunnett’s multiple comparison test with untreated controls (no antibody) serving as the comparison data set (*p < 0.05, **p < 0.01, ***p < 0.001, ****p < 0.0001). **(A)** Siplizumab significantly decreased CD69^+^ T cells on day 2 at 0.1–10 µg/ml (p ≤ 0.0490) and on day 4 at 10 µg/ml siplizumab (p = 0.0351). **(B)** HLA-DR expression on T cells was significantly inhibited on days 2, 4, and 7 at 0.1 µg/ml (p = 0.0104; p = 0.0373; p = 0.0008), 1 µg/ml (p = 0.0059; p = 0.0189; p = 0.0005), and 10 µg/ml (p = 0.0021; p = 0.0190; p = 0.0005) siplizumab. **(C)** Alemtuzumab significantly increased CD69^+^ T cells after 1 day of allogeneic MLR at 0.01–10 µg/ml (p ≤ 0.0091). On days 2, 4, and 7, no dose-dependent change in CD69 expression was observed. **(D)** Alemtuzumab significantly increased HLA-DR^+^ T cells on day 1 at 0.01–10 µg/ml (p ≤ 0.0344), on day 2 at 0.1 µg/ml (p = 0.0167) and 1 µg/ml (p = 0.0157), as well as on day 4 at 1 µg/ml (p = 0.0261) and 10 µg/ml (p = 0.0010). No significant differences were detected on day 7. **(E)** A significant increase in CD69^+^ T cells was detected after 1, 2, and 4 days of allogeneic MLR in samples treated with 0.001 µg/ml (p ≤ 0.0467), 0.01 µg/ml (p ≤ 0.0024), 0.1 µg/ml (p ≤ 0.0017), 1 µg/ml (p ≤ 0.0007), and 10 µg/ml rATG (p < 0.0001). After 7 days, rATG induced a significant increase in CD69 expression on T cells at 0.01–10 µg/ml (p < 0.0001). **(F)** rATG induced a modest but statistically significant increase in HLA-DR^+^ T cells after 1 day of allogeneic MLR at 0.01–10 µg/ml (p ≤ 0.0056) as well as after 2 days at 0.01–1 µg/ml (p < 0.0159). In contrast, a decrease in HLA-DR expression in rATG-treated samples was noted at 0.01–10 µg/ml; however, only 0.01 µg/ml (p = 0.0170) and 10 µg/ml (p = 0.0048) were significant. **(G)** Representative histograms showing CD69 expression on T cells after 1 day of MLR (upper panel) and HLA-DR expression after 7 days of MLR (lower panel) in samples treated with 10 µg/ml siplizumab or 10 µg/ml rATG.

Given that T cell proliferation and differentiation tended to occur during later time points (after day 4), activation marker expression (CD69 and HLA-DR) on T cells in allogeneic MLRs was analyzed at baseline and after 1, 2, 4, and 7 days of alogeneic MLR to detect potential early effects of mAb treatment on T cell activation (**Figure 6** and **Supplementary Tables S10 and S11**).

The percentage of CD69^+^ T cells in untreated controls increased from a baseline level of circa 1.25% to 6.5%–7% on days 4 and 7, respectively. HLA-DR expression on T cells increased from approximately 3.40% HLA-DR+ T cells at baseline to circa 30.52% after 7 days of allogeneic MLR. Siplizumab induced a significant decrease in the percentage of CD69^+^ T cells after 2 days of allogeneic MLR at 0.1 µg/ml (p = 0.0490), 1 µg/ml (p = 0.0279), and 10 µg/ml (p = 0.0216). Significant inhibition of T cell CD69 expression was maintained on day 4 in samples treated with 10 µg/ml siplizumab (p = 0.0351). Additionally, HLA-DR expression on T cells was significantly inhibited after 2, 4, and 7 days of allogeneic MLR in samples treated with 0.1 µg/ml (p ≤ 0.0373), 1 µg/ml (p ≤ 0.0189), and 10 µg/ml (p ≤ 0.0190) siplizumab. In contrast, Alemtuzumab significantly increased the percentage CD69^+^ T cells and HLA-DR^+^ T cells after 1 day of allogeneic MLR at 0.01 µg/ml (p = 0.0091; p = 0.0344), 0.1 µg/ml (p = 0.0002; p < 0.0001), 1 µg/ml (p = 0.0013; p = 0.0012), and 10 µg/ml (p = 0.0002; p = 0.0189). Alemtuzumab induced a significant increase in HLA-DR expression after 2 days of allogeneic MLR at 0.1 µg/ml (p = 0.0167) and 1 µg/ml (p = 0.0157). Increased HLA-DR expression at 10 µg/ml on day 2 trended toward significance (p = 0.0731). After 4 days HLA-DR expression on T cells remained significantly elevated in allogeneic MLRs treated wth 1 µg/ml (p = 0.0261) and 10 µg/ml (p = 0.0010) Alemtuzumab. A significant increase in CD69^+^ T cells was detected after 1, 2, and 4 days of allogeneic MLR in samples treated with 0.001 µg/ml (p ≤ 0.0467), 0.01 µg/ml (p ≤ 0.0024), 0.1 µg/ml (p ≤ 0.0017), 1 µg/ml (p ≤ 0.0007), and 10 µg/ml rATG (p < 0.0001). After 7 days, rATG induced a significant increase in CD69 expression on T cells at 0.01 µg/ml, 0.1 µg/ml, 1 µg/ml, and 10 µg/ml (p < 0.0001 for all concentrations). A decrease in HLA-DR expression in rATG-treated samples was noted at 0.01–10 µg/ml; however only, 0.01 µg/ml (0.0170) and 10 µg/ml (p = 0.0048) reached significance.

**Figure 7:**
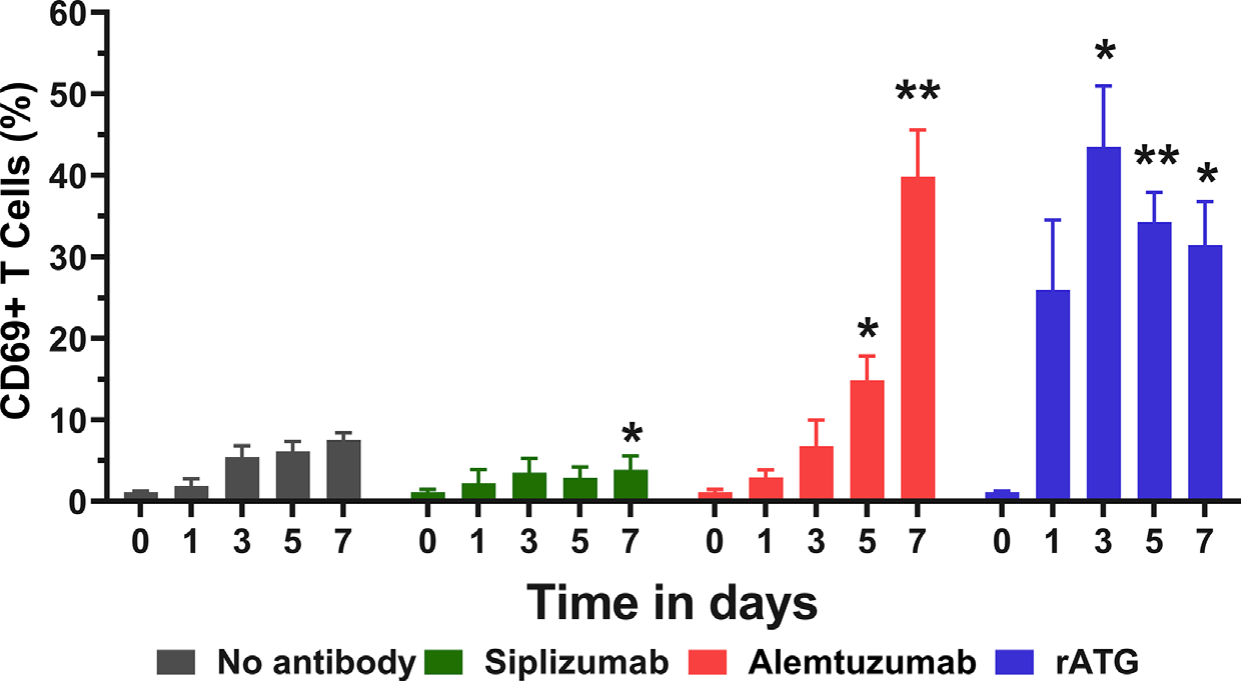
CD69 expression in pure T cell culture. Untouched T cells were purified using negative magnetic-activated cell sorting and kept in autologous culture for up to 7 days. Data displayed as mean percentage of CD69+ T cells +SD (N = 4). Data were analyzed using two-way ANOVA followed by Dunnett’s multiple comparison test (*p < 0.05, **p < 0.01). Siplizumab significantly reduced CD69 expression after 7 days of autologous culture (p = 0.0260), but not on previous time points. In contrast, Alemtuzumab significantly elevated CD69+ T cells after 5 (p = 0.0328) and 7 (p = 0.0057) days of autologous culture. rATG induced a significant increase in CD69 expression on T cells after 3 (p = 0.0232), 5 (p = 0.0052), and 7 (p = 0.0327) days.

To investigate the effects of target antigen-binding in isolation, untouched T cells were isolated using negative magnetic bead-selection and kept in autologous culture with or without the addition of antibody drugs. Expression of CD69 on T cells was measured at baseline (day 0) and after 1, 3, 5, and 7 days of autologous culture (**Figure 7** and **Supplementary Table S12**). Siplizumab induced a significant decrease in CD69+ T cells after 7 days of autologous culture (p = 0.0260). Alemtuzumab significantly elevated CD69 expression on T cells after 5 (p = 0.0328) and 7 (p = 0.0057) days. Similarly, rATG significantly elevated CD69 expression on T cells after 3 (p = 0.0232), 5 (p = 0.0052), and 7 (p = 0.0327) days of autologous T cell culture.

### NK Cell Activation

**Figure 8:**
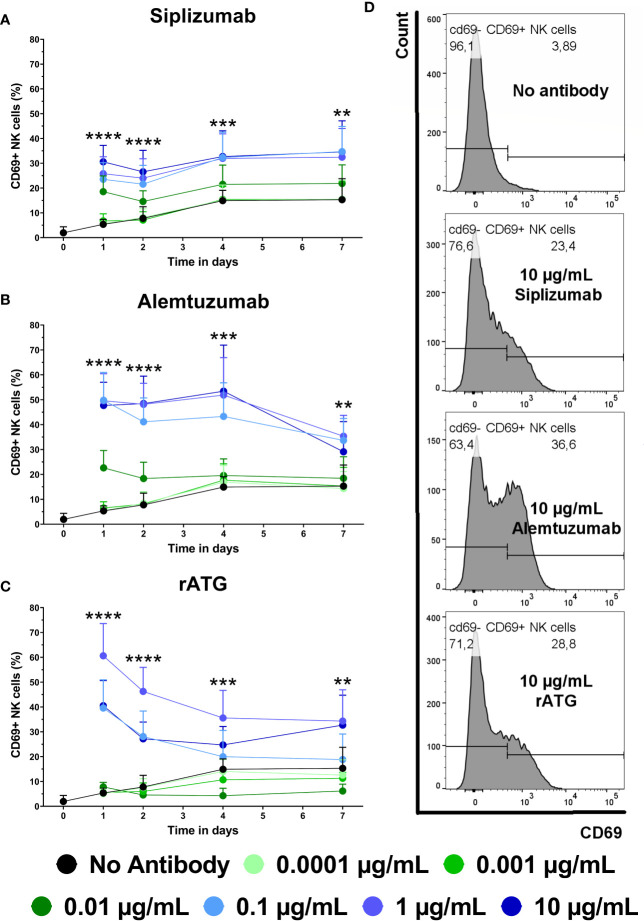
The effect of siplizumab **(A)**, Alemtuzumab **(B)**, and rabbit anti-thymocyte globulin (rATG) **(C)** and on CD69 expression on NK cells in allogeneic mixed lymphocyte reaction (MLR). Expression of CD69 was analyzed at baseline (day zero) and on days 1, 2, 4, and 7. Data is displayed as the mean of all data points (N = 9 donor pairs) ± SD. NK cells were identified as CD3^−^ CD56^+^. Data were analyzed using two-way ANOVA followed by Dunnett’s multiple comparison test with untreated controls (no antibody) serving as the comparison data set (**p < 0.01, ***p < 0.001, ****p < 0.0001). **(A)** Siplizumab significantly increased CD69^+^ NK cells at 0.001–10 µg/ml (p ≤ 0.0284) on day 1 and at 0.01–10 µg/ml (p ≤ 0.0010; p ≤ 0.0377; p ≤ 0.0248) on days 2, 4, and 7 of allogeneic MLR. **(B)** Alemtuzumab significantly increased CD69 expression on NK cells on day 1 at 0.001–10 µg/ml (p ≤ 0.0248), on day 2 at 0.01–10 µg/ml (p ≤ 0.0024), on day 4 at 0.1–10 µg/ml (p ≤ 0.0007), and on day 7 at 0.1 µg/ml (p = 0.0003) and 1 µg/ml (p = 0.0076). **(C)** rATG significantly increased CD69^+^ NK cells at 0.01–10 µg/ml (p ≤ 0.0102) on day 1, 0.1–10 µg/ml (p ≤ 0.0022) on day 2, 1 µg/ml (p = 0.0022) and 10 µg/ml (p = 0.0266) on day 4, as well as 1 µg/ml (0.0017) and 10 µg/ml (p = 0.0038) on day 7 of allogeneic MLR. rATG inhibited CD69 expression on NK cells on day 4 at 0.001 µg/ml (0.0034) and 0.01 µg/ml (p = 0.0001). **(D)** Representative histograms showing CD69 expression on NK cells after 1 day of MLR in samples treated with 10 µg/ml siplizumab or 10 µg/ml rATG.

CD69 is an early activation marker on T and NK cells. As depletory mAbs can induce ADCC *via* NK cells, NK cell activation induced by mAb treatment was investigated. NK cells were defined as CD3^−^ CD56^+^ lymphocytes and CD69 expression on NK cells was analyzed at baseline (day 0) and after 1, 2, 4, and 7 days of allogeneic MLR (**Figure 8** and **Supplementary Table S13**).

The percentage of CD69+ NK cells in allogeneic MLR increased from on average 1.95% at baseline to 15.29% on day 7. On day 1, siplizumab induced a significant increase in CD69^+^ NK cells at 0.001 µg/ml (p = 0.0284), 0.01 µg/ml (p = 0.0001), and 0.1–10 µg/ml (p < 0.0001). On days 2, 4, and 7 of allogeneic MLR, siplizumab significantly increased CD69^+^ NK cells at 0.01 µg/ml (p ≤ 0.0377), 0.1 µg/ml (p ≤ 0.0100), 1 µg/ml (p ≤ 0.0248), and 10 µg/ml (p ≤ 0.0115). Similarly, Alemtuzumab significantly increased CD69 expression on NK cells on day 1 at 0.001 µg/ml (p = 0.0248), 0.01 µg/ml (p = 0.0001), and 0.1–10 µg/ml (p < 0.0001), on day 2 at 0.01 µg/ml (p = 0.0024) and 0.1–10 µg/ml (p < 0.0001), on day 4 at 0.1 µg/ml (p = 0.0005), 1 µg/ml (p = 0.0003) and 10 µg/ml (p = 0.0007), as well as on day 7 at 0.1 µg/ml (p = 0.0003) and 1 µg/ml (p = 0.0076). In comparison, rATG significantly increased CD69^+^ NK cells at 0.01–10 µg/ml (p ≤ 0.0102) on day 1, 0.1–10 µg/ml (p ≤ 0.0022) on day 2, 1–10 µg/ml (p ≤ 0.0266) on day 4 as well as 1–10 µg/ml (p ≤ 0.0038) on day 7 of allogeneic MLR. Interestingly, rATG inhibited CD69 expression on NK cells on day 4 at 0.001 µg/ml (0.0034) and 0.01 µg/ml (p = 0.0001).

### Target Antigen Expression

Heterogenous target antigen expression on T and NK cell subpopulations may lead to certain subpopulations being more or less affected by mAb treatment. Therefore, target antigen expression on T and NK cell subpopulations was measured *via* flow cytometry. Moreover, target antigen expression was investigated on both resting and activated PBMC to detect any potential up- or downregulation associated with cell activation.

**Figure 9:**
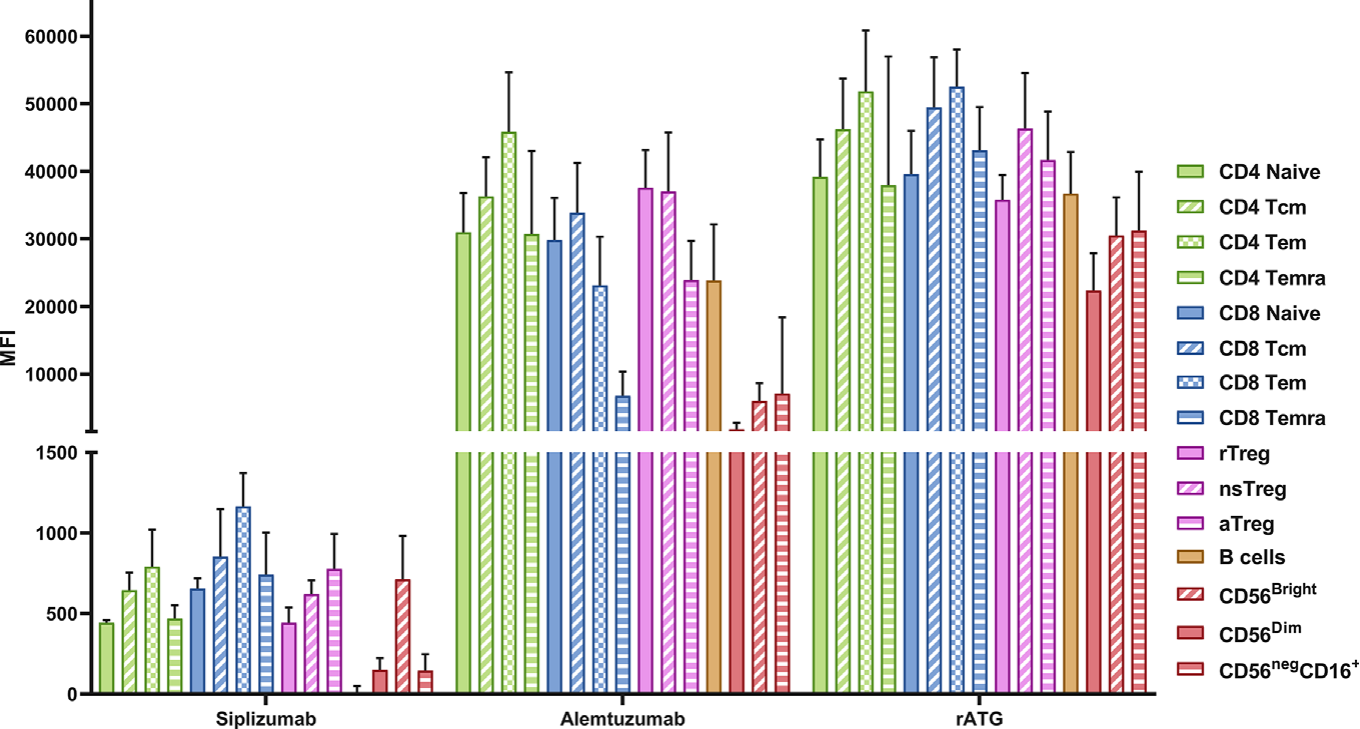
Target antigen expression on PBMC. Data is displayed as the average median fluorescent intensity (MFI) ± SD (N = 5). PBMC were stained using the following drugs: siplizumab (Target antigen CD2), Alemtuzumab (CD52) and rATG (Thymocyte antigens). After removal of unbound antibody, samples were stained with a secondary anti-human IgG antibody and MFI for the secondary antibody was recorded, representing the expression of the respective transplant antigens. Tcm, central memory T cells; Tem, effector memory T cells; Temra, effector-memory CD45RA^+^ T cells; rTreg, resting regulatory T cells; nsTreg, non-suppressive Treg; aTreg, activated Treg; CD56^Bright^, CD56^bright^ NK cells; CD56^Dim^, CD56 ^dim^ NK cells; CD56^neg^ CD16^+^, CD56^−^ CD16^+^ NK cells.

Expression of target antigens was measured on resting PBMC using antibodies investigated in this study and secondary anti-IgG antibodies (**Figure 9** and **Supplementary Table S14**). All MFI values for T and NK cells were adjusted for background signal *via* an isotype control (Rituximab). Tn express CD2 at lower levels than Tmem, with the highest expression seen on Tem. CD56^Bright^ NK cells had a higher expression of CD2 compared to CD56^Dim^ and CD56^Neg^CD16^+^ NK cells. CD52 was highly expressed on cell types investigated although somewhat lower on NK cells and CD8+ Temra compared to other T cell populations and B cells. CD25 is relatively highly expressed on CD4^+^ T cells and Tregs in particular. rATG is a polyclonal antibody mixture with some of the most common clones binding CD2, CD5, CD8, CD11a, and CD18 (19). Clones were present and reactive to all cell types investigated, rATG also had somewhat higher antigen density on Tmem compared to naïve.

**Figure 10:**
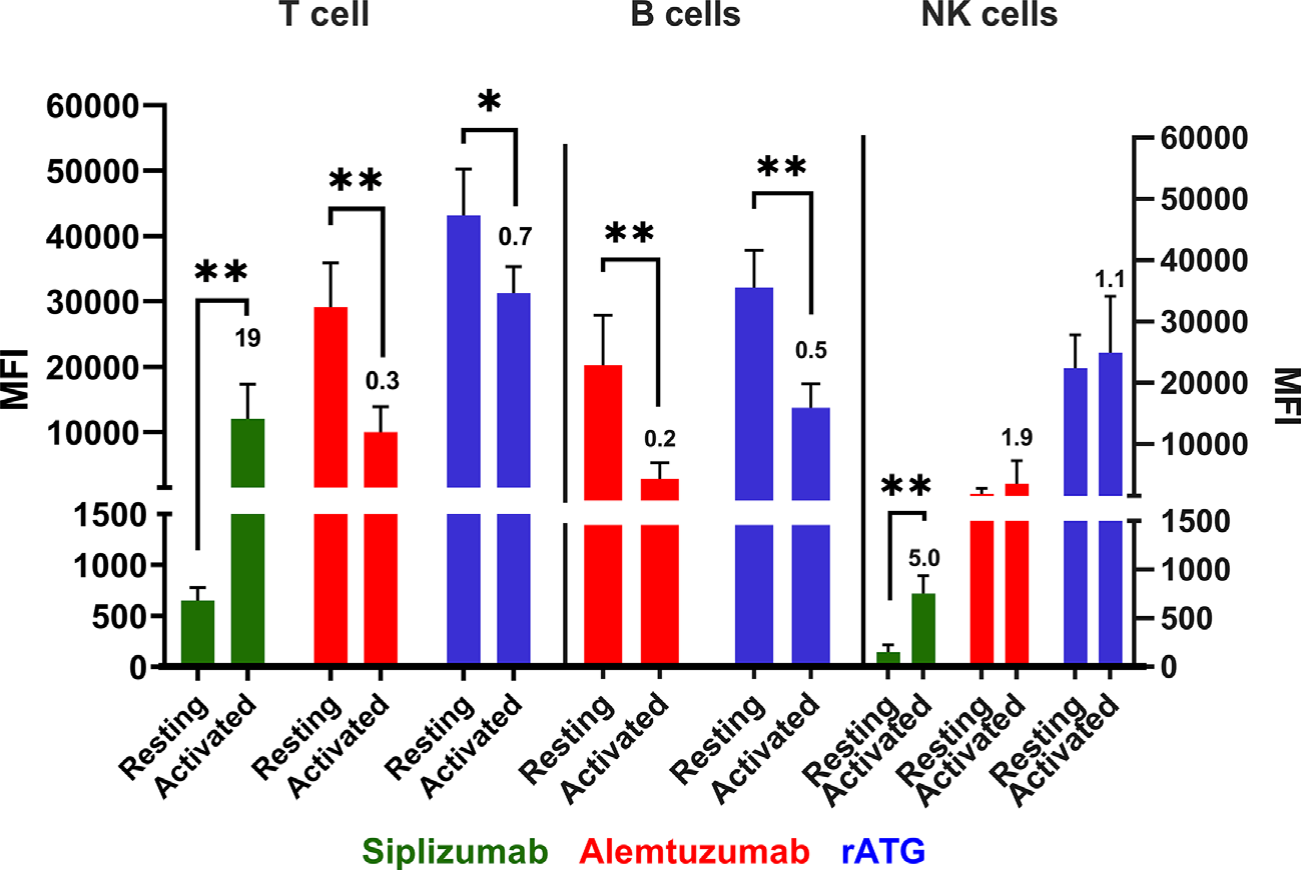
Antibody binding to PBMC populations in a resting state or activated state. Data is displayed as average median fluorescent intensity (MFI) ± SD (N = 5). Ratio of activated divided by resting MFI is displayed above bars for activated cells. PBMC were examined either in a resting state immediately following isolation or after activation using CD28/CD3 beads for 48 h. Only antibodies with relevant expression level (mean MFI over 50) were plotted for the respective cell type. Data were analyzed using paired two-sided t test comparing pre- and post-activation MFI (*p < 0.05, **p < 0.01). A significant increase in siplizumab binding to T (19-fold; p = 0.0083) and NK cells (five-fold; p = 0.0013) was measured upon PBMC activation. Alemtuzumab binding to T (0.3-fold; p = 0.0032) and B (0.2-fold; p = 0.0045) cells declined significantly. Lastly, rATG binding to T (0.7-fold; p = 0.0188) and B (0.5-fold; p = 0.0050) cells decreased significantly following activation.

Following PBMC activation using anti-CD3/anti-CD28 microbeads target antigen expression was investigated once more (**Figure 10** and **Supplementary Table S14**). CD2 expression (siplizumab binding) was significantly upregulated on activated T [19-fold; p = 0.0083 (two-sided paired t test)] and NK cells (five-fold; p = 0.0013) relative to resting equivalents. Upon activation, Alemtuzumab binding to T (0.3-fold; p = 0.0032) and B (0.2-fold; p = 0.0045) cells declined significantly. Lastly, rATG binding to T (0.7-fold; p = 0.0188) and B (0.5-fold; p = 0.0050) cells decreased significantly following activation.

**Table 1** summarizes the data on effector functions and effects in allogeneic MLR obtained for each investigated antibody.

**Table 1:** Summary of the characteristics of different tested compounds.

	Siplizumab	Alemtuzumab	rATG	References
**Target antigen**	CD2	CD52	Thymocyte antigens	
**Type of protein**	mAb	mAb	pAb	
**ADCC/ADCP**	+	+^1^	+^1^	**Figure 1**
**CDC**	0	+^1^	+^1^	**Figure 1**
**T cell depletion**	+	+	+	**Figure 3**
**T cell activation**	−	+	+	**Figures 6** and **7**
**T cell proliferation**	−	0	+	**Figure 2**
**Tn enrichment**	+	+/−	0	**Figure 4**
**Treg enrichment**	+	+/−	+	**Figure 5**
**NK cell activation**	+	+	+	**Figure 8**

ADCC, antibody-dependent cell-mediated cytotoxicity; ADCP, antibody-dependent cell phagocytosis; CDC, complement-dependent cytolysis; mAb, monoclonal antibody; pAb, polyclonal antibody; +, increases given effect; −, inhibits given effect/read-out; 0, no change/does not induce given effect; +/−, inconclusive assay results.

^1^See text for references.

## Discussion

This study presented a mechanistic characterization of siplizumab and a direct comparison of siplizumab with Alemtuzumab and rATG in allogeneic MLR. Overall, siplizumab induces multiple effects in allogeneic MLR that are desirable in a transplantation or autoimmunity context. Notably, siplizumab inhibits T cell activation, reduces T cell proliferation and enriches Tn and *bona fide* Tregs. While Alemtuzumab and rATG induced some of the same effects, no tested agent other than siplizumab induced all in combination.

The *in vitro* nature of this study was a major limitation. The effects of a drug observed in an *in vitro* assay may not reflect *in vivo* effects that manifest in the complex physiological and immunological environment of patients. Furthermore, this study contained a relatively small sample size which means that MLR results may not perfectly reflect that average response that would be observed across the whole population. Additionally, PBMC were obtained from healthy donors who may not display immunological responses representative of kidney transplant recipients who usually suffer from autoimmune disorders.

The mechanistic assays used herein to test for ADCC, ADCP, and CDC allow for comparison of what effector mechanisms may contribute to depletion *via* siplizumab or other depletory antibody agents assessed in this study. The fact that siplizumab-mediated depletion stems from ADCC and ADCP but not CDC (**Figure 1**) may prove advantageous in settings where depletion of T and NK cells is desired but the risk for cytokine release syndrome is to be minimized. Complement fixation and associated release of anaphylatoxins like C3a and C5a could increase inflammatory signaling which is undesirable in transplant patients or patients suffering from autoimmune disorders (20). Notably, the importance of ADCP for antibody-mediated target cell depletion may be underestimated in an MLR setting. Depletion of target cells *in vivo* is commonly observed starting shortly after infusion. Substantial activation of ADCC in such a short time is unlikely to explain all of the antibody-mediated depletion *in vivo* and thus the relative abundance of phagocytes *in vivo* (e.g., Kupffer cells in the liver) may explain the rapid clearance of target cells from peripheral blood (21).

Siplizumab, Alemtuzumab and rATG had a depletory effect on T cells as part of their mechanism of action (MoA; **Figure 3**). Siplizumab reduced T cell count only after T cell proliferation had commenced. In contrast, T cell depletion mediated by rATG and Alemtuzumab was observed before any cell division was detected. As siplizumab induced significant NK cell activation, T cell depletion relative to untreated controls after 7 days of allogeneic MLR likely derived from both inhibition of T cell proliferation and ADCC-mediated T cell depletion. However, as siplizumab did not seem to mediate depletion during the early stages of allogeneic MLR, activation and differentiation-mediated upregulation of CD2 expression may be needed to achieve a sufficient number of target molecules per cell to induce depletion. As NK cells are both a target of siplizumab and the main mediator of ADCC in an MLR setting, fast depletion of target cells using siplizumab *in vivo* (8) can likely be explained by ADCP-mediated depletion. Indeed, the dominance of ADCP in anti-CD2 mAb-mediated depletion is in agreement with previous pre-clinical findings (22). Relatively mild T cell depletion by rATG in this study can likely be explained by the absence of CDC and ADCP in allogeneic MLR. Serum used for MLR was heat-inactivated and PBMC contain few functionally mature phagocytes, leaving ADCC and apoptosis induction as the two main depletory effector mechanisms. As previously described, rATG mainly depletes T cells through CDC, ADCP and apoptosis (18).

Siplizumab prevented the expansion of Tmem and enriched Tn and Tregs which is in line with previously published studies (6, 7). Enrichment of Tn and Tregs *via* siplizumab likely resulted from both inhibition of T cell proliferation and relative depletion of Tmem.

While both rATG and siplizumab induced a relative enrichment of Tregs among proliferated CD4^+^ T cells when compared to untreated controls, FoxP3 promoter methylation data in this study showed that a substantial amount of activation-induced FoxP3 expression occured in untreated controls and in samples treated with rATG but not in samples treated with siplizumab (**Table 1**). As rATG consists of a complex mixture of polyclonal antibodies against thymocyte antigens including the TCR-CD3 complex and other costimulatory molecules, it is possible that some clones in rATG may have inhibitory and/or Treg-inducing properties similar to clinically used costimulatory blockers while other clones may have a mitogenic effect, similar to, e.g., OKT3 (19). Indeed, pure T cell cultures confirmed a mitogenic effect of rATG on T cells. Consequently, enrichment of Tregs *via* siplizumab resulted in cells which are more phenotypically committed (*bona fide*) Tregs. Interestingly, anti-CD2 antibodies are among the most abundant clones in rATG (19). Considering observed effects using rATG or siplizumab in this study, anti-CD2 antibodies in rATG may be responsible for a considerable portion of the immune modulatory effects seen using rATG. Thus, siplizumab enables a more selective mediation of these effects while avoiding mitogenic effects induced by other antibodies in rATG. Indeed, treatment of PBMC cultures with rATG depleted of anti-TCR-CD3, anti-CD28, and anti-CD2 clones lowered expression of CD69, CD25, and FoxP3 on CD4 T cells relative to PBMC cultures treated with normal rATG (19). Future studies should investigate to what extent enrichment of Tregs by siplizumab derives from less depletion of Tregs relative to other T cell subpopulations and/or CD2/LFA3 CoB.

Previous evidence has shown that Tregs enriched by siplizumab are CD45RA^−^ and mostly aTregs, despite their relatively high CD2 expression (Podesta et al., 2019). Therefore, Tregs may be more resistant to siplizumab-mediated depletion than other T cell subpopulations and/or less reliant on CD2/LFA3 costimulation for activation and differentiation. The fact that siplizumab enriches aTregs despite their high CD2 expression further suggests that depletion of CD2^+^ cells may not be the sole mechanism of action of siplizumab. The exact mechanisms by which siplizumab enriches Tregs remain incompletely characterized and should be investigated more deeply in future research. Notably, it should be investigated whether Fc-receptor-mediated crosslinking of siplizumab-bound CD2 has effects on T cell activation, proliferation, differentiation and interaction with APCs which are independent of Fc receptor-mediated depletion and CD2/LFA3 CoB.

When using depletory antibodies, the phenotypes of both depleted and non-depleted cells are important to consider. Reconstitution from lymphopenic states is achieved by both thymic and bone marrow output of naïve lymphocytes as well as homeostatic proliferation of non-depleted lymphocytes (23). Depletion elicits a decline in cell count which leads to an increased amount of certain cytokines that normally deliver survival signals per remaining peripheral lymphocyte. This induces a state of “spontaneous” proliferation which is largely proportional to the degree of lymphopenia (23). Murine models have shown that Tmem seem to undergo homeostatic proliferation more readily than Tn (24). Homeostatically proliferated T cells originating from Tmem seem to have more potent effector functions than ones derived from Tn, even though both assume a memory phenotype during homeostatic proliferation (25). Especially in older human cohorts which are known to have low thymic output (26), homeostatic proliferation may constitute a dominant mechanism of reconstitution. Consequently, the importance of what cell types remain after antibody-mediated depletion may have been underappreciated in the past.

While non-depletory antibody agents would avoid the potential problem of homeostatic proliferation of Tmem and depletion of pathogen-reactive T cells, they do not eliminate alloreactive cells. Thus, while these agents may contribute to preventing rejection, they are unlikely to make significant contributions to immunological engraftment as they merely prevent activation of alloreactive T cells instead of actively modelling the T cell repertoire toward selective tolerance. Given that CD2 is upregulated on Tmem and upon T cell activation, siplizumab may be an optimal depletory agent to use for skewing the post-depletion T cell repertoire toward Tn and Treg phenotypes.

T cell malfunction characterizes many autoimmune and chronic inflammatory pathologies, for example multiple sclerosis (MS), rheumatoid arthritis (RA), and type-1 diabetes. In these cases, the use of a T cell depletory agent could be of great interest. Investigators using the anti-CD2 biologic Alefacept for treatment of type-1 diabetes have reported promising results (27). Alemtuzumab is a currently used treatment of MS but targeting T cells in an isolated manner might reduce unwanted side effects. Treating MS with isolated T cell depletion could be questioned as auto-antibodies has been found to play a role in MS and B cell depletory agents are used as treatment for the disease to great success (28). Still, combination of siplizumab with a B cell depletory agent may achieve similar depletion with more favorable immune modulation. While targeting T cells in RA might seem promising, several candidates targeting CD4 and CD5 have failed to show clinical results (29). In these studies autoreactive Tmem were spared, highlighting the need for a treatment able to target more depletion-resistant Tmem subpopulations (30). To summarize, a drug with immunomodulatory and T cell depleting properties could play an important role in various autoimmune diseases and should be investigated further.

In conclusion, each of the tested antibody agents has displayed a unique functional profile. When considering desired characteristics for an antibody agent used during organ transplant induction therapy or treatment of autoimmune diseases (deletion of alloreactive/disease-mediating Tmem, enrichment of Tn and Treg, relative preservation of lymphocytes that mediate pathogen-immunity), siplizumab has a particularly attractive mechanistic profile. None of the tested agents is likely to create an optimal immunological environment in patients by itself. However, the informed use of these agents as part of more elaborate treatment protocols can contribute to minimizing the risk of rejection and need for immunosuppression long-term as well as maximizing the chance of patients developing durable immune tolerance.

## Data Availability Statement

The raw data supporting the conclusions of this article will be made available by the authors, without undue reservation.

## Author Contributions

CB and FS carried out experiments and wrote the first manuscript. All authors contributed to the article and approved the submitted version.

## Conflict of Interest

All authors are employees of ITB MED AB. EB and DB own shares in ITB MED AB.

## Abbreviations

ADCC, Antibody-dependent cell-mediated cytotoxicity; ADCP, Antibody-dependent cell phagocytosis; APC, Antigen-presenting cell; CDC, Complement-dependent cytolysis; CoB, Costimulatory blockade; FcγR, Fc γ receptor; HLA, Human leukocyte antigen; mAb, Monoclonal antibody; mIS, Maintenance immunosuppression; MLR, Mixed-lymphocyte reaction; MoA, Mechanism of action; PBMC, Peripheral blood mononuclear cells; rATG, Rabbit anti-thymocyte globulin; Tcm, Central memory T cells; Tem, Effector memory T cells; Temra, Terminally differentiated effector memory T cells; Tn, Naïve T cells; Tmem, Memory T cells; Treg, Regulatory T cells; VPD, Violet proliferation dye.
