# Supplementary material for: Siplizumab, an Anti-CD2 Monoclonal Antibody, Induces a Unique Set of Immune Modulatory Effects Compared to Alemtuzumab and Rabbit Anti-Thymocyte Globulin In Vitro
Source: Front Immunol. 2020 Nov 11;11:592553. doi: 10.3389/fimmu.2020.592553 (PMC7686512; doi:10.3389/fimmu.2020.592553)
Supplement: Supplementary file 1 [file Table_1.docx]

**Supplementary figures**


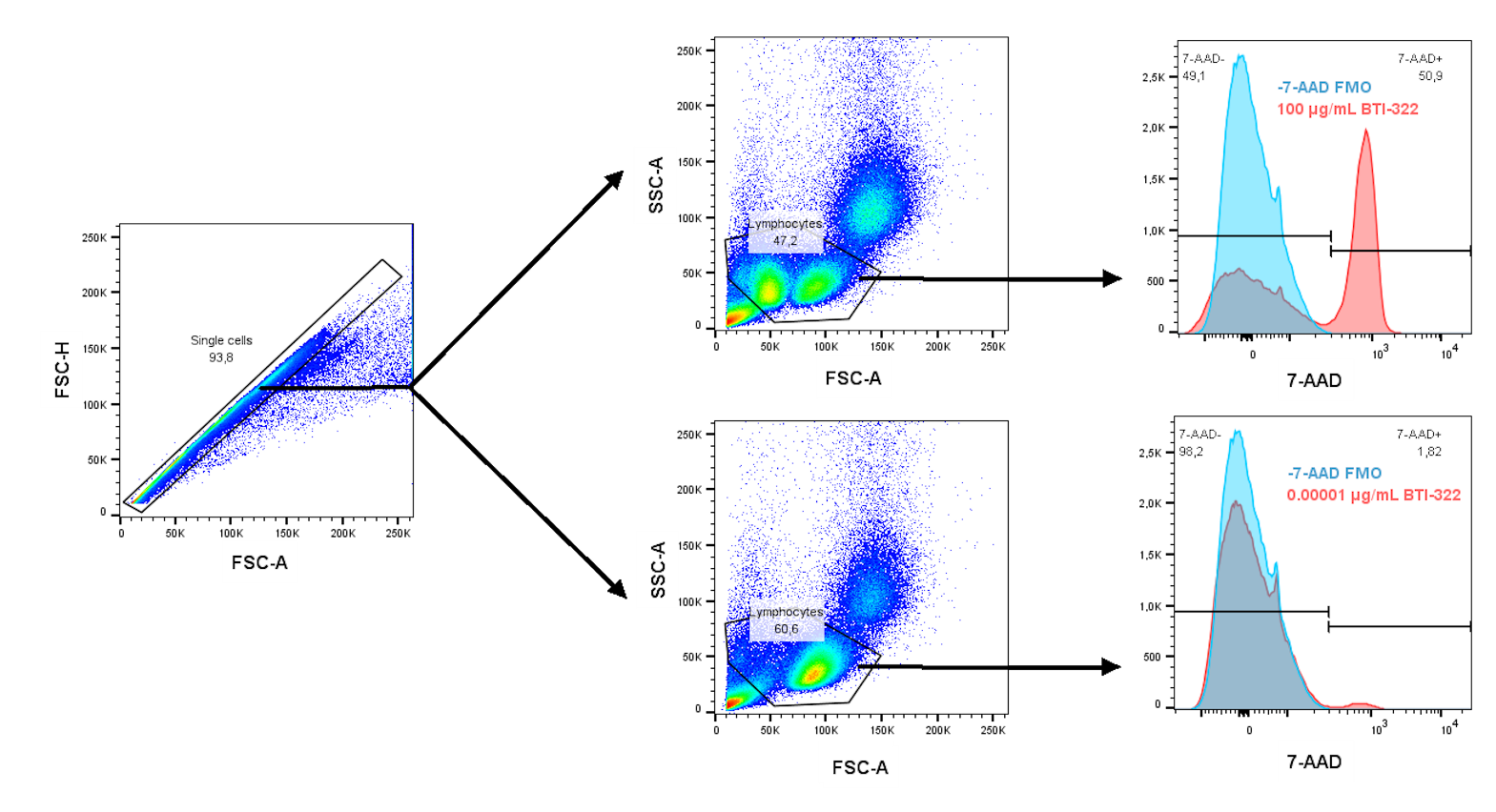


**Supplementary figure S1. CDC assay gating strategy.** Gating strategy for assessment of antibody-mediated CDC. Single cells were identified using forward scatter area and forward scatter height. Lymphocytes were identified using forward scatter area and side scatter area. Lysed cells were identified as staining positive for 7-AAD using a fluorescence minus one control (FMO).


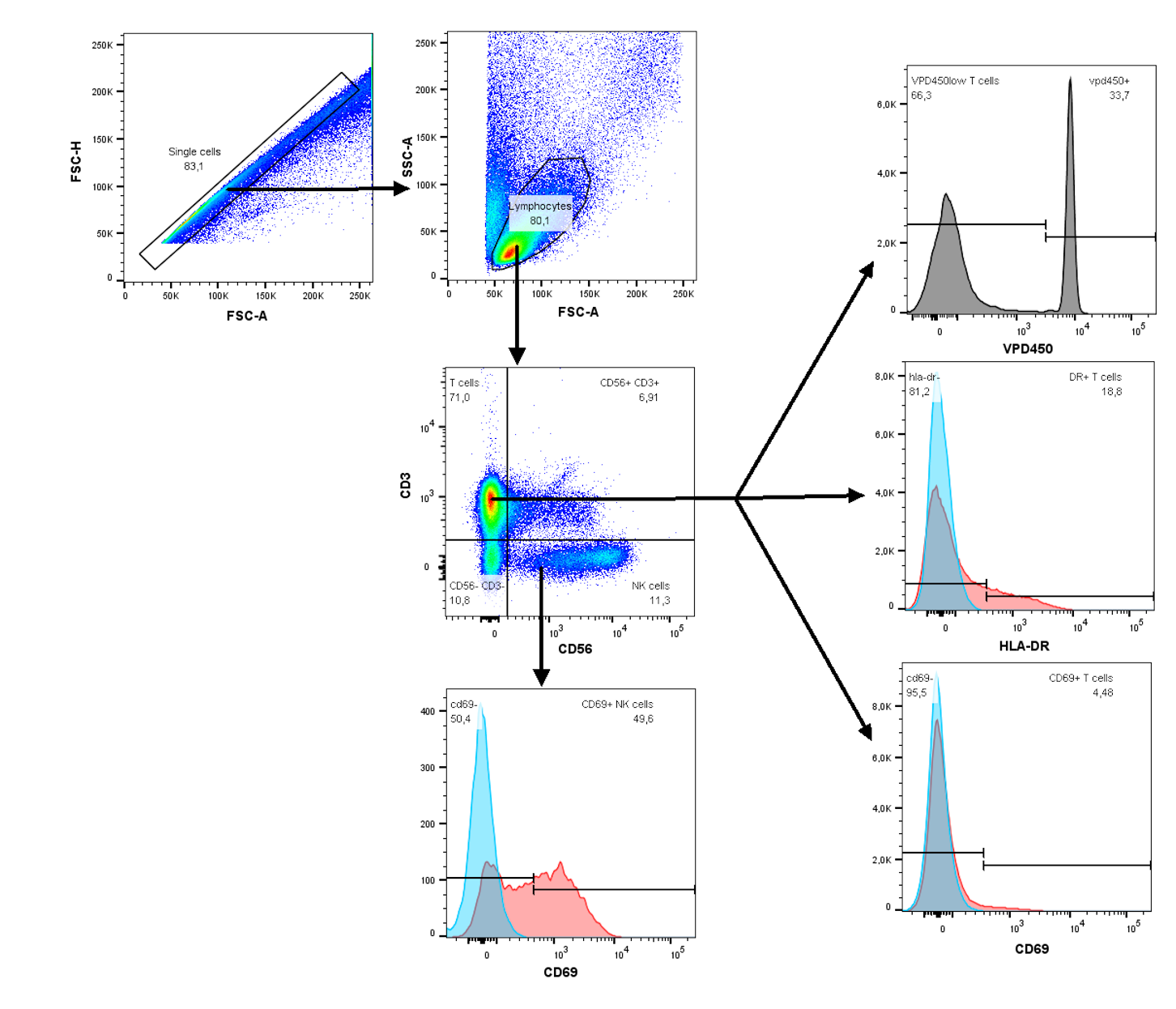


**Supplementary figure S2. T and NK cell activation gating strategy.** Gating strategy for assessment of T and NK cell activation. Single cells were identified using forward scatter area (FSC-A) and forward scatter height (FSC-H). Lymphocytes were identified using forward scatter area (FSC-A) and side scatter area (SSC-A). T cells and NK cells were identified as CD3^+^ CD56^-^ and CD3^-^ CD56^+^, respectively. CD69^+^ and HLA-DR^+^ T and NK cells were identified using fluorescence minus one controls (blue). Proliferated T cells were identified as displaying no or weak violet proliferation dye 450 fluorescence intensity (VPD450low).


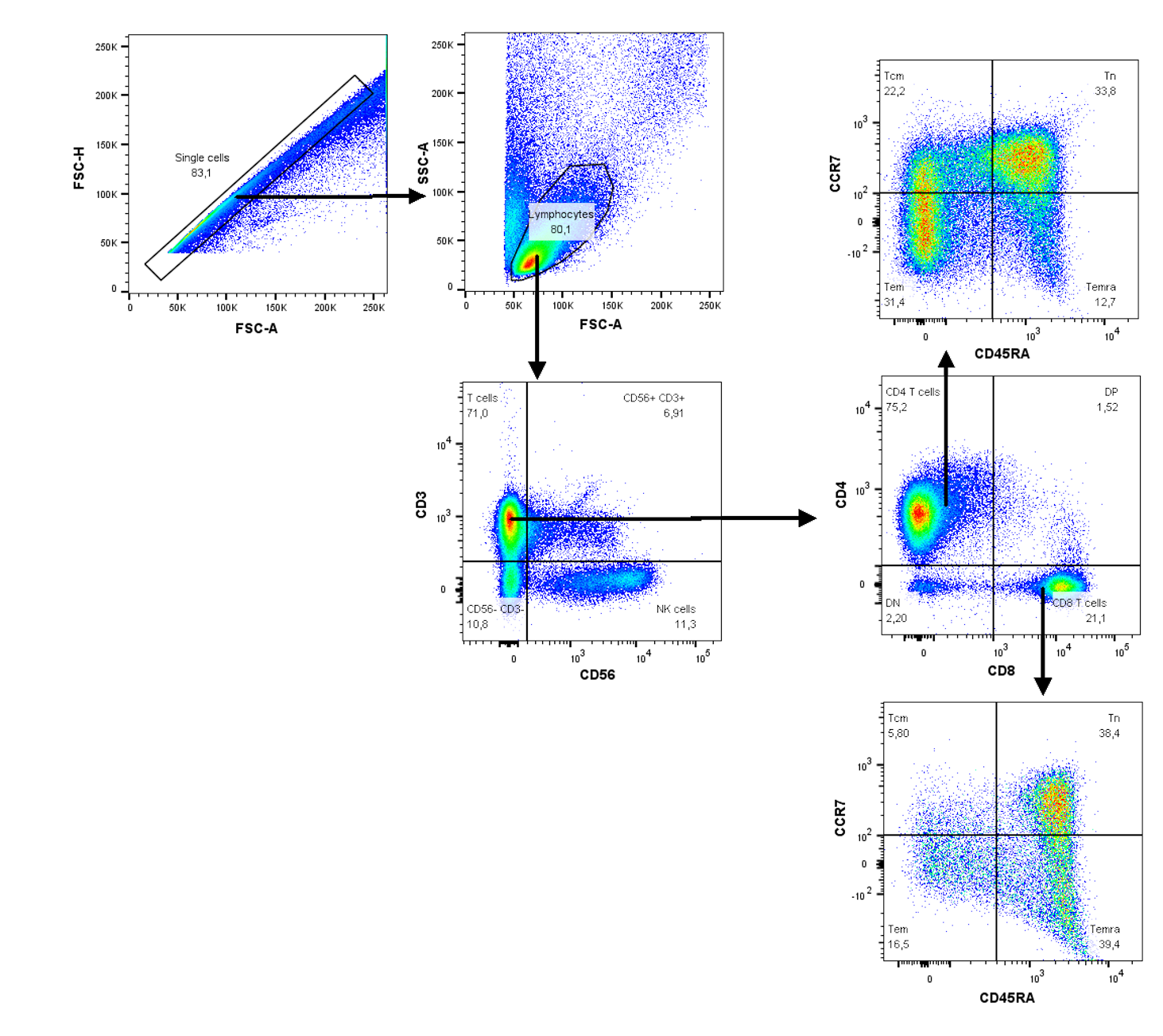


**Supplementary figure S3. Memory and naïve T cell gating strategy.** Single cells were identified using forward scatter area (FSC-A) and forward scatter height (FSC-H). Lymphocytes were identified using forward scatter area (FSC-A) and side scatter area (SSC-A). T cells were identified as CD3^+^ CD56^-^. CD4 and CD8 T cells were divided into central memory T cells (Tcm; CCR7^high^ CD45RA^-^), naïve T cells (Tn; CCR7^high^ CD45RA^+^), terminally-differentiated effector memory T cells (Temra; CCR7^-^ CD45RA^+^) and effector memory T cells (Tem; CCR7^-^ CD45RA^-^).


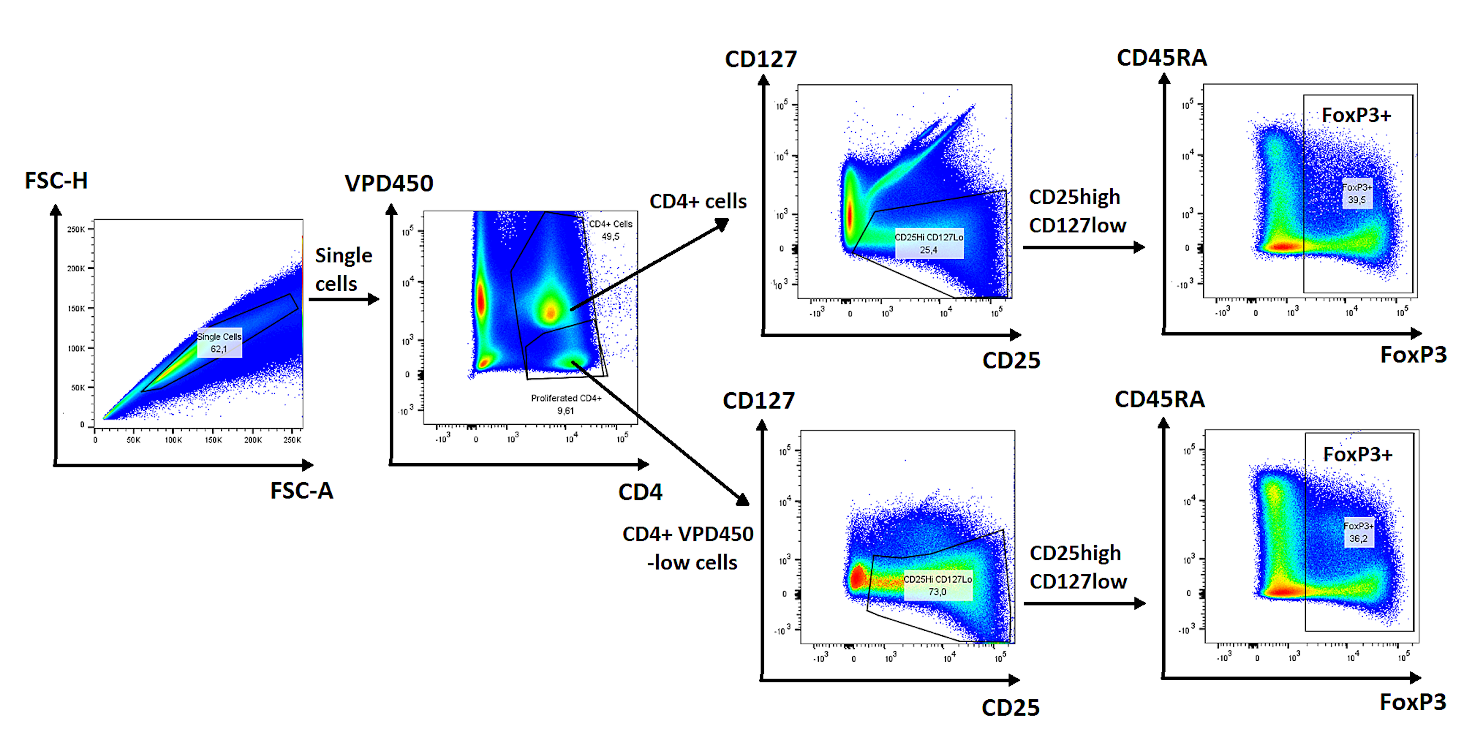
**Supplementary figure S4. Regulatory T cell (Treg) gating strategy.** Single cells were identified using forward scatter area (FSC-A) and forward scatter height (FSC-H). CD4^+^ T cells were identified as CD4^+^ cells and divided into VPD450high/low (resting/proliferating CD4^+^ T cells). Treg gating was conducted for both all and VPD450low CD4^+^ cells. Tregs were identified as CD4^+^ CD127^-^ CD25^+^ FoxP3^+^. Tregs were measured as percent of grandparent population (All or VPD450low CD4^+^ T cells, respectively).

**Supplementary tables**

**Table S1. Normalized luminescence in Fc γ receptor I reporter bioassay.** Data displayed as mean normalized luminescence +SD (N=3). Highest concentration used was 6.67 nM or 1 µg/mL (Molecular weight 150 kDa).

**Table S2. Normalized luminescence in Fc γ receptor IIA reporter bioassay.** Data displayed as mean normalized luminescence +SD (N=3). Highest concentration used was 666.7 nM or 100 µg/mL for Siplizumab and 66.7 nM or 10 µg/mL (Molecular weight 150 kDa).

**Table S3. Normalized luminescence in Fc γ receptor IIIA reporter bioassay.** Data displayed as mean normalized luminescence +SD (N=3). Highest concentration used was 6.67 nM or 1 µg/mL (Molecular weight 150 kDa).

**Table S4. Lymphocyte lysis in CDC assay.** Data displayed as mean percentage if 7-AAD+ lymphocytes +SD (N=3). Highest concentration used was 666.7 nM or 100 µg/mL (Molecular weight 150 kDa).

**Table S5. T cell proliferation in allogeneic mixed lymphocyte reaction.** Data displayed as mean percentage of proliferated T cells +SD (N=9 donor pairs). Data were analyzed using two-way ANOVA followed by Dunnett’s multiple comparison test with untreated controls (no antibody) serving as the comparison data set (* p<0.05, ** p<0.01, *** p<0.001, **** p<0.0001).

**Table S6. Normalized T cell count in allogeneic mixed lymphocyte reaction.** Data displayed as mean normalized T cell count +SD (N=9 donor pairs). Data were analyzed using two-way ANOVA followed by Dunnett’s multiple comparison test with untreated controls (no antibody) serving as the comparison data set (* p<0.05, ** p<0.01, *** p<0.001, **** p<0.0001).****

**Table S7. Naïve CD4^+^ T cells in allogeneic mixed lymphocyte reaction.** Data displayed as mean percentage of naïve (CCR7^high^ CD45RA^+^) cells among CD4^+^ T cells +SD (N=9 donor pairs). Data were analyzed using two-way ANOVA followed by Dunnett’s multiple comparison test with untreated controls (no antibody) serving as the comparison data set (* p<0.05, ** p<0.01, *** p<0.001, **** p<0.0001).

**Table S8. Naïve CD8^+^ T cells in allogeneic mixed lymphocyte reaction.** Data displayed as mean percentage of naïve (CCR7^high^ CD45RA^+^) cells among CD8^+^ T cells +SD (N=9 donor pairs). Data were analyzed using two-way ANOVA followed by Dunnett’s multiple comparison test with untreated controls (no antibody) serving as the comparison data set (* p<0.05, ** p<0.01, *** p<0.001, **** p<0.0001).

**Table S9. Changes in the percentage of CD4 T cells with a regulatory phenotype in allogeneic MLR.** Data displayed as mean percentage ± SD (N=12). Data were analyzed using two-way ANOVA followed by Dunnett’s multiple comparison test (* p<0.05, ** p<0.01, *** p<0.001, **** p<0.0001).

| % of total CD4+ | **Day** | **Drug** | **Control** |  | **Siplizumab** | **rATG** |
| --- | --- | --- | --- | --- | --- | --- |
|  | 7 | Mean | 7,39 |  | 7,06 | 18,18^****^ |
|  |  | SD | 1,12 |  | 2,65 | 5,25 |
|  | 10 | Mean | 8,85 |  | 13,01^*^ | 28,56^****^ |
|  |  | SD | 3,61 |  | 2,68 | 6,46 |
|  | 14 | Mean | 8,18 |  | 17,93^****^ | 29,08^****^ |
|  |  | SD | 4,47 |  | 2,44 | 5,49 |
| % of proliferated CD4+ | 7 | Mean | 14,62 |  | 30,02^***^ | 37,23^****^ |
|  |  | SD | 6,70 |  | 10,37 | 11,01 |
|  | 10 | Mean | 15,70 |  | 46,19^****^ | 46,56^****^ |
|  |  | SD | 6,86 |  | 12,62 | 11,73 |
|  | 14 | Mean | 13,46 |  | 49,51^****^ | 43,27^****^ |
|  |  | SD | 8,32 |  | 15,86 | 10,77 |
| % FoxP3 promoter methylation | 0 | Mean | 21.92 |  |  |  |
|  |  | SD | 14.27 |  |  |  |
|  | 7 | Mean | 56.88 |  | 29.38^*^ | 37.98 |
|  |  | SD | 6.88 |  | 11.15 | 9.99 |
|  | 14 | Mean | 43.94 |  | 20.66 | 30.18 |
|  |  | SD | 5.22 |  | 14.63 | 10.64 |

**Table S10. CD69^+^ T cells in allogeneic mixed lymphocyte reaction.** Data displayed as mean percentage of CD69^+^ cells among T cells +SD (N=9 donor pairs). Data were analyzed using two-way ANOVA followed by Dunnett’s multiple comparison test with untreated controls (no antibody) serving as the comparison data set (* p<0.05, ** p<0.01, *** p<0.001, **** p<0.0001).

**Table S11. HLA-DR^+^ T cells in allogeneic mixed lymphocyte reaction.** Data displayed as mean percentage of HLA-DR^+^ cells among T cells +SD (N=9 donor pairs). Data were analyzed using two-way ANOVA followed by Dunnett’s multiple comparison test with untreated controls (no antibody) serving as the comparison data set (* p<0.05, ** p<0.01, *** p<0.001, **** p<0.0001).

**Table S12. CD69^+^ T cells in pure T cell culture.** Data displayed as mean percentage of CD69^+^ cells among T cells +SD (N=4 donors). Data were analyzed using two-way ANOVA followed by Dunnett’s multiple comparison test with untreated controls (no antibody) serving as the comparison data set (* p<0.05, ** p<0.01, *** p<0.001, **** p<0.0001).

**Table S13. CD69^+^ NK cells in allogeneic mixed lymphocyte reaction.** Data displayed as mean percentage of CD69^+^ cells among NK cells +SD (N=9 donor pairs). Data were analyzed using two-way ANOVA followed by Dunnett’s multiple comparison test with untreated controls (no antibody) serving as the comparison data set (* p<0.05, ** p<0.01, *** p<0.001, **** p<0.0001).

**Table S14: Target antigen expression on T cell subpopulations, B cells and NK cell subpopulations.** Data is displayed as average median fluorescent intensity ± SD.

| **Resting**  **cells** | **T cells** | **CD4 Tn** | **CD4 Tcm** | **CD4 Tem** | **CD4 Temra** | **CD8 Tn** | **CD8 Tcm** | **CD8 Tem** | **CD8 Temra** | **rTreg** | **nsTreg** | **aTreg** | **B cells** | **NK cells** | **CD56^Bright^** | **CD56^Dim^** | **CD56^neg^CD16^+^** |
| --- | --- | --- | --- | --- | --- | --- | --- | --- | --- | --- | --- | --- | --- | --- | --- | --- | --- |
| **Siplizumab** | 652 (± 126) | 443 (± 17) | 645 (± 109) | 7912 (± 229) | 469 (± 82) | 656 (± 63) | 853 (± 296) | 1165(± 207) | 740 (± 263) | 442 (± 97) | 621,1 (± 86,6) | 778 (± 217) | < 50 | 151 (± 74.0) | 713 (± 270) | 151 (± 74) | 145 (± 103) |
| **Alemtu-**  **zumab** | 29148 (± 6703) | 30950 (± 5823) | 36274 (± 5786) | 45851 (± 1019) | 30709 (± 12295) | 29800 (± 6260) | 33860 (± 7389) | 23141 (± 7173) | 6791 (± 3583) | 37558 (± 5598) | 37035 (± 8734) | 23938 (± 5755) | 23861 (± 8284) | 1830 (± 945) | 6009 (± 2660) | 1830 (± 945) | 7132 (± 11276) |
| **rATG** | 43159 (± 7063) | 39154 (± 5563) | 46234 (± 7507) | 51840 (± 9023) | 37949 (± 19037) | 39574 (± 6412) | 49485 (± 7424) | 52528 (± 5491) | 43084 (± 6418) | 35794 (± 3659) | 46350 (± 8215) | 41656 (± 7186) | 36675 (± 6160) | 22359 (± 5507) | 30500 (± 5621) | 22359 (± 5507) | 31231 (± 8700) |
| **Activated cells** | **T cells** | **CD4 Tn** | **CD4 Tcm** | **CD4 Tem** | **CD4 Temra** | **CD8 Tn** | **CD8 Tcm** | **CD8 Tem** | **CD8 Temra** | **rTreg** | **nsTreg** | **aTreg** | **B cells** | **NK cells** | **CD56^Bright^** | **CD56^Dim^** | **CD56^neg^CD16^+^** |
| **Siplizumab** | 12030** (± 5309) | 13630 (± 4178) | 11134 (± 4965) | 10765 (± 7454) | 10771 (± 6570) | 12365 (± 6384) | 9774 (± 4837) | 2906 (± 2660) | 1971 (± 243) | 12460 (± 2342) | 10914 (± 2969) | 11434 (± 3135) | < 50 | 752** (± 184) | 3465 (± 1675) | 752 (± 184) | 1012 (± 256) |
| **Alemtu-**  **zumab** | 10019** (± 3876) | 12145 (± 5412) | 13881 (± 5838) | 12436 (± 5408) | 8140 (± 4646) | 7264 (± 4197) | 5682 (± 1223) | 5909 (± 2351) | 3831 (± 3565) | 10620 (± 3821) | 11088 (± 4647) | 10635 (± 3552) | 5112** (± 2650) | 3518 (± 3784) | 1703 (± 1039) | 3518 (± 3783) | 1377 (± 212) |
| **rATG** | 31265* (± 4030) | 32419 (± 3217) | 32029 (± 4607) | 21722 (± 6414) | 21788 (± 11624) | 31740 (± 6103) | 28706 (± 9608) | 5815 (± 8010) | 16641 (± 2334) | 40056 (± 4964) | 31836 (± 3960) | 30492 (± 4931) | 16863** (± 3962) | 24889 (± 9189) | 24787 (± 3118) | 24889 (± 9188) | 13128 (± 2399) |
